# Preterm birth skews the developmental trajectory of myeloid-derived suppressor cells in the first 48 hours of life: single-cell transcriptomics and inferred intercellular communication

**DOI:** 10.1186/s10020-026-01459-8

**Published:** 2026-03-27

**Authors:** Miguel Hernández-Ríos, Jaimar Rincon, Leandro Balzano-Nogueira, Valerie Polcz, Dayuan Wang, Whitman Wiggins, Christine Rodhouse, Athina Yoham, Feifei Xiao, Ricardo Ungaro, Marvin Dirain, Lyle Moldawer, Robert Maile, Guoshuai Cai, Philip Efron, Shawn Larson

**Affiliations:** 1 Sepsis and Critical Illness Research Center, Department of Surgery, University of Florida College of Medicine, Gainesville, Florida USA; 2 Division of Pediatric Surgery, Department of Surgery, University of Florida College of Medicine, P.O. Box 100119, 1600 SW Archer Road 3261, University 0-0019, Gainesville, Florida 32610-0019 USA; 3 Department of Pathology, Immunology, and Laboratory Medicine, University of Florida, Gainesville, Florida USA; 4 Department of Biostatistics, University of Florida Colleges of Medicine and Public Health and Health Sciences, Gainesville, Florida USA

**Keywords:** Preterm neonates, Myeloid-derived suppressor cells, Developmental trajectory, Cell communication, Single-cell RNA sequencing

## Abstract

**Background:**

Prematurity is a leading cause of neonatal and childhood mortality, with infections driving early deaths. Innate immunity provides frontline defense after birth and is shaped in part by myeloid-derived suppressor cells (MDSCs). The role of MDSCs in regulation of neonatal immunity, especially in the context of prematurity, remains elusive. We sought to understand the transcriptional landscape of neonatal immune myeloid regulators, specifically differences between preterm and full-term neonates. Insight into specific cellular networks could help understand how to skew preterm MDSCs’ development towards classical immunotolerant and anti-microbial mechanisms.

**Methods:**

This cross-sectional study used single-cell RNA sequencing to characterize the neonatal MDSC transcriptional landscape, developmental trajectories, and predicted signaling networks within 48 h after birth. Peripheral blood mononuclear cells were isolated from 7 preterm neonates (< 36 weeks gestational age), 6 full-term neonates (> 37 weeks), and 6 healthy adult (21–45 years old, control). Primary exposure was premature birth and neonatal intensive care hospitalization from a single-center, academic tertiary care hospital between 2023 – 2024.

**Results:**

Among ~ 339,000 cells, preterm neonates exhibited enrichment of polymorphonuclear MDSCs (10.6% ± 5.3%) vs full-term (2.6% ± 1.3%) and adults (0.4% ± 1.3%). Trajectory analysis identified a prematurity-associated differentiation branch characterized by inflammatory signaling, mitochondrial stress, and heightened protein-translation programs, distinct from a conserved, tolerogenic MDSC trajectory present across ages. Cell-communication modeling showed intensified outgoing and incoming signaling via ADGRE, RESISTIN, TGF-β, ANNEXIN, and ICAM networks. Antigen presentation signatures suggested preserved MHC-I output and diminished MHC-II interactions in neonates, with increased MHC-I input to preterm polymorphonuclear MDSCs.

**Conclusions:**

Prematurity is associated with early divergence of MDSC maturation toward an inflammatory and metabolically stressed PMN-MDSC state with altered immune communication. These findings identify cellular mechanisms that may contribute to the heightened tissue damage and infection susceptibility of preterm neonates and highlight MDSC signaling as a potential target for early-life immunomodulatory interventions.

**Supplementary Information:**

The online version contains supplementary material available at 10.1186/s10020-026-01459-8.

## Introduction

Preterm birth is a significant global health concern, affecting approximately 10% of all births worldwide and representing a leading cause of neonatal mortality (Ohuma et al. 2023). Premature birth occurs during a critical stage of development, abruptly separating the fetus from the protective intrauterine environment and exposing immature organ systems to substantial physiological changes (e.g., blood pressure variability, hormone fluctuations, oxygen exposure), as well as potentially harmful external factors including inflammation, infection, and pharmacologic interventions (Haren et al. 2024). Survivors of preterm birth are at increased risk of long-term complications, including neurodevelopmental impairments, chronic health conditions, and a heightened susceptibility to sepsis and necrotizing enterocolitis, largely due to impaired immune responses (Sarda et al. 2021).

After birth, the neonatal immune system faces a complex challenge as it must clear pathogens while maintaining tolerance to self-antigens and supporting microbial colonization. Although immune regulation in preterm neonates remains poorly understood, emerging evidence suggests that lymphoid organs and immune effector cells are fully developed and functional by the second trimester, capable of mounting effective immune responses and even acquiring some degree of immune memory in utero (Vidal and Menon 2023). However, it is increasingly recognized that the preterm immune system is primarily adapted for intrauterine survival under adverse conditions (Stolfi et al. 2025; Humberg et al. 2020; Ramos et al. 2023) and is vulnerable to poor outcomes when confronted with the immunological demands of early extrauterine life.

MDSCs are a heterogeneous population of myeloid cells with potent immunosuppressive functions. In humans, two major subsets have been characterized: polymorphonuclear-MDSCs (PMN-MDSCs) defined classically as CD66b^+^CD11b^+^CD33^+^HLA-DR^low/−^, and monocytic-MDSCs (M-MDSCs), defined as CD14^+^CD11b^+^CD33^+^HLA-DR^low/−^. MDSCs expand during cancer, chronic inflammation, pregnancy, and the neonatal period (Dorhoi and Plessis 2018; Li et al. 2021; Pang et al. 2023). Their immunosuppressive activity involves inhibition of T and NK cells functions via multiple mechanisms, including PD-L1 expression and production of reactive oxygen species (ROS), nitric oxide (NO), and arginase-1 (Arg-1) (Dorhoi and Plessis 2018; Brauner et al. 2025). Additionally, MDSCs produce anti-inflammatory cytokines such as IL-10 and TGF-β and promote the expansion of regulatory T cells (Tregs) with enhanced suppressive capacity, supporting a tolerogenic immune environment (Polcz et al. 2023).

Postnatal immune development (including both innate and adaptive immune function) depends heavily on environmental cues and cellular interactions. However, the unique molecular characteristics of neonatal MDSCs and their cellular crosstalk, particularly in preterm neonates, remain poorly defined. It is unclear whether MDSCs in prematurity represent a distinct myeloid cell subpopulation compared to those in full-term neonates or healthy adults.

Here, we apply single-cell transcriptomics to define how prematurity reshapes early-life MDSC differentiation and immune communication within the first 48 h after birth. We test the hypothesis that preterm birth induces an alternative MDSC developmental trajectory, characterized by expansion of polymorphonuclear MDSCs with altered metabolic and signaling programs. By integrating pseudotime inference and ligand–receptor modeling, we identify molecular pathways and intercellular communication circuits that nominate MDSCs as key drivers of immune dysregulation in prematurity and potential targets for early-life immunomodulatory strategies.

## Methods

### Study enrollment

This single center, prospective study was conducted in a tertiary care, academic research hospital (UF Health Shands Hospital, USA) and aimed to characterize neonatal MDSCs and investigate their functional differences across age at the transcriptomic level. Preterm neonates were enrolled if born with a gestational age (GA) between 26 to 36 weeks (exclusion criteria: congenital or chromosomal anomalies incompatible with life or requiring emergent surgical intervention [including ECMO], renal anomalies, receipt of exchange blood transfusion within 48 h of life, being born to a mother with type 1 diabetes, or a history of immunodeficiency/immunosuppression). The second cohort included healthy full-term neonates with a gestational age ≥ 37 weeks (exclusion criteria: congenital abnormalities, admission to the NICU or congenital heart ICU, being born to a mother with type 1 diabetes, or a history of immunodeficiency/suppression). The third cohort included healthy adult participants (21—45 years; exclusion criteria: pregnancy, ongoing corticosteroid or immunosuppressive therapy, organ dysfunction, HIV infection, autoimmune disease or cancer). The study was approved by the University of Florida Institutional Review Board (IRB), and written informed consent was obtained from the parents or guardians of neonates, and from all healthy adult participants.

### Blood collection and sample preparation

Blood was collected in EDTA collection tubes (Becton Dickinson, USA) within the first 48 h after birth from preterm neonates (250–300 µl), and at day of life 1 from full-term neonates (500 µl). For healthy adult participants, 1 ml of whole blood was collected via venipuncture. Clinical and research data collection were entered into a web-based electronic case report form created on the REDCap™ platform managed by the University of Florida Clinical and Translational Science Institute. Maternal demographics, indications for delivery, and postnatal data (including demographics) were collected and are outlined in Table 1 and Table S1. Following sample collection, the PBMC was enriched by density centrifugation (Ficoll-Paque™ PLUS, Cytiva, USA) within 3 h of blood collection.

**Table 1:** Cohort characteristics of single-cell RNA sequencing subjects

Patient characteristics	Preterm Neonates (*n* = 7)	Full Term Neonates (*n* = 6)	Healthy Adults (*n* = 6)
*Demographics*			
Median age week gestational age or years (range)	29.7 (27.6–31.9) w GA	39.4 (37.3–40.7) w GA	31.5 (25–41) years
Median birthweight (range)	1415 (745–1710) g	3320 (1903–2237) g	N/A
Median corrected GA at T1 blood collection, (range)	30.1, (28.1–32.1) w GA	N/A	N/A
Median corrected GA at discharge, (range)	37.7, (36.8–44.7) w GA	N/A	N/A
Male, n (%)	3 (43)	3 (50)	2 (33)
Apgar scores (mean score 1 min/5 min)	5/7	8/9	N/A
*Ethnicity (mean, %)*			
Hispanic/Latino	0	2, (33)	1, (17)
Not Hispanic/Latino	6, (86)	4, (67)	5, (83)
Not reported	1, (14)	0	0
*Race (mean, %)*			
White/Caucasian	2, (29)	6, (100)	5, (83)
Black/African American	4, (57)	0	0
American Indian/Alaskan native	0	0	0
Hawaiian native/Pacific Islander	0	0	0
Asian	0	0	1, (17)
Other	0	0	0
Not reported	1, (14)	0	0
*Maternal prenatal history*			
Prenatal care	6 (86) Yes, 1 (14) unknown	6 (100) Yes	N/A
Auto-immune disease	1 (14) Hashimoto Thyroiditis	0	N/A
Tobacco use	0	0	
Toxicology screen	0	0	N/A
Preeclampsia	2 (29)	1	N/A
Magnesium exposure	3 (43) Yes	0	N/A
Betamethasone exposure	3 (43) Yes	0	N/A
Gestational Diabetes	0	1 (17) Yes	N/A
Chorioamnionitis	1 (14) Yes	0	N/A
Serology	0	1 (17) (+ GBS)	N/A
PPROM	2 (29) Yes	0	N/A
C-section (mean)	6 (86) Yes	1 (17) No	N/A
*Post-natal hospital course*			
EOS (+ BCx < = 72 h of life)	0	0	N/A
LOS (+ BCx > 72 h of life)	1 (14) Yes	0	N/A
Antibiotic for 5 + days for presumed infection	3 (43) Yes	6 (100) No	N/A
IVH	2 (29) Yes	0	N/A
NEC	0	0	N/A

T1: 24–48 h after birth; *w* Weeks, *GA* Gestational age, *g* Grams, *GBS* Gram positive beta-streptococcus, *EOS* Early onset sepsis, *LOS* Late onset sepsis, *BCx* Blood culture, *IVH* Intra-ventricular hemorrhage, *NEC* Necrotizing enterocolitis

### Flow cytometry

Thirty-seven preterm and 25 full-term neonates were enrolled for this experiment, along with 12 healthy adults. Cohort characteristics are provided in Table S1, and antibodies used for surface staining are listed in Key Resources. For surface staining, PBMCs (~ 1 × 10^6^ cells) were incubated for 15 min at 4 °C with an antibody cocktail targeting MDSC-associated surface markers, including: HLA-DR-APC/Fire 750, CD11b-PE, CD33-FITC, CD14-APC, CD66b-Bv421. Following staining, cells were washed with PBS and acquired on a ZE5 Cell Analyzer (Bio-Rad Laboratories, Hercules, CA). Data were analyzed using FlowJo™ v10.8 (BD Biosciences, Franklin Lakes, NJ). To quantify circulating MDSCs, cells were initially gated on HLA-DR^low/−^CD11b^+^CD33^+^ populations (Fig. S1). These were further delineated based on CD14 and CD66b expression to identify specific MDSC subsets: M-MDSCs (HLA-DR^low/−^CD11b^+^CD33^+^CD14^+^) and PMN-MDSCs (HLA-DR^low/−^CD11b^+^CD33^+^CD11b^+^CD66b^+^) (Darden et al. 2021). Compensation was performed using compensation beads (BD Biosciences, Franklin Lakes, NJ). Statistical significance was assessed using the Kruskal–Wallis test, followed by Dunn’s multiple comparisons test with Bonferroni correction.

### Library preparation and sequencing

Fresh PBMC suspensions were filtered with a 30-micron MACS® SmartStrainers (Miltenyi Biotec) and resuspended in PBS containing 0.04% bovine serum albumin. After cell counting, samples were adjusted to 1,000 cells/µl and loaded onto the Chromium chips following the 10X Genomics protocol for single-cell 3’ kits, version 3 (10X Genomics, USA).

#### Single-cell RNA-sequencing read preprocessing and quality control metrics

Gene expression profiling was performed using 10 × Genomics 3’ v3 chemistry and sequenced on an Illumina NovaSeq X platform, targeting ~ 10,000 cells per sample (Zheng et al. 2017). The BCL Convert (Illumina) software suite was used to process base calls into FASTQ files, which were checked for quality control aberrations using FastQC v0.11.7 (S.A 2010). A spliced + intronic (i.e., splici) reference transcriptome was generated from the hg38 reference genome (Gaidatzis et al. 2015). Reads were pseudo-aligned to the reference transcriptome with alevin-fry v0.8.1; USA mode was used for the gene expression reads to provide separate quantifications of spliced, unspliced, and ambiguous mRNA abundance (Srivastava et al. 2020, 2019; He et al. 2022).

#### Single-cell RNA-sequencing data processing

Downstream data processing and analysis were performed primarily in R v4.4.1, with some additional processes being written in Python v3.10 as required (Python n.d.); (Guido 2022). The total mRNA counts were used as input throughout the analysis. Empty droplets were identified and removed using the emptyDrops algorithm implemented in DropletUtils.v1.18.1 (Griffiths et al. 2018; Lun et al. 2019). Cells with an estimated false discovery rate (FDR) of < 0.01 were kept for each sample. Next, the percentage of spliced reads coming from mitochondrial genes was computed for each cell, and cells with greater than 5% mitochondrial DNA were excluded. The final merged dataset was composed of 26,430 genes and 339,251 cells.

#### Single-cell RNA-sequencing dimensionality reduction, normalization and data integration

The read counts for each gene were log-normalized and transformed prior to dimensionality reduction; hierarchical clustering was based on Ward’s distance (Murtagh and Legendre 2014). Next, all samples were integrated by the Harmony package v1.2.0 (Korsunsky et al. 2019) and the first 50 principal components were used as input to generate UMAPs (McInnes, et al. 2018). Lastly, a first round of clusters were generated via Louvain modularity optimization using a resolution of 0.9 and the cosine distance with the number of nearest neighbors set to 100 Blondel 2008.

#### Single-cell RNA-sequencing annotation

After clustering, multiple methods were executed to perform the cell annotation in SingleR v2.4.1 and Azimuth 0.4.6.9004. Both packages perform cell identification by matching individual cells to a reference dataset of known cell types; thus, a set of informative genes that distinguish different cell types was generated. SingleR was employed to build a predictive model using selected genes from the reference dataset via a single-sample gene set enrichment analysis (ssGSEA) approach. Each cell in the target dataset was annotated with a predicted cell type label from the reference dataset (Aran et al. 2019). A total of 5 different reference datasets were used to annotate the cells: Blueprint ENCODE data, 259 RNA-seq samples of pure stroma and immune cells (Joost and Hendrik 2013; Dunham et al. 2012); Database Immune Cell Expression Data (DICE), 1561 bulk RNA-seq samples of sorted cell populations (Schmiedel et al. 2018); Human Primary Cell Atlas Data, a community that has annotated millions of human cells from many different tissues (Mabbott et al. 2013); Monaco Immune Data, 114 bulk RNA-seq samples of sorted immune cell populations that can be found in GSE10701110 (Monaco et al. 2019); Novershtern Hematopoietic Data, comprising 211 bulk human microarray samples of sorted hematopoietic cell populations that can be found in GSE2475911 (Novershtern et al. 2011). Azimuth was also used to annotate, utilizing a publicly available healthy human PBMC reference with 161,764 cells (Hao et al. 2021). Cells were annotated by consensus of 5 out of 6 (> 0.80) annotation procedures, which allowed a more homogenous cell annotation. To further refine the myeloid compartment, a supervised approach was performed to improve the annotation. The expression of myeloid-specific gene markers was assessed within clusters identified through a second round of Louvain modularity optimization across a range of resolutions (0.6 to 2, step size of 0.1). This supervised refinement was performed not only to increase annotation accuracy, but also to allow the identification of specialized or transitional populations such as MDSCs that are not explicitly represented in commonly used reference atlases, and therefore, require expert-guided interpretation based on canonical marker expression patterns and biological context.

#### Differential expression analysis, functional enrichment, and pathway analysis

Differentially expressed genes (DEG) were identified after stratification by cell type using a mixed-effects model implemented in Julia (Bezanson et al. 2017). In all analyses, samples from the preterm and full-term neonates were considered as the comparison group while those from healthy adults were considered as the reference group. The mixed-effects model was employed to assess the differential expression of genes across conditions, while controlling individual variability. This approach provided a robust framework for identifying DEG in relatively low sample size datasets. The p-values were adjusted for multiple testing using FDR. All age groups comparison were performed using an adjusted *p*-value < 0.01. For visualization and ranking purposes, volcano plots were adjusted using the apeglm method from DESeq2 (v1.44.0). This approach pulls low-confidence estimates toward zero, improving interpretability without affecting p-values or adjusted p-values.

Enriched canonical pathways were identified using Ingenuity Pathway Analysis (IPA QIAGEN Inc. Germantown, MD, https://digitalinsights.qiagen.com/IPA). Statistical significance was assessed using Fisher’s exact test and adjusted for multiple comparisons using the Benjamini–Hochberg correction. Only genes with significantly altered expression (FDR < 0.05 and absolute log_2_ fold change ≥ 1) were included in the analysis. Pathway activity was inferred using IPA’s activation z-score algorithm, where a z-score ≥ 2.00 indicates predicted activation and z-score ≤ −2.00 indicates predicted inhibition.

#### Single-cell RNA-sequencing trajectory inference

Preprocessed integrated datasets were analyzed using Monocle3 package (v1.3.7) (Cao et al. 2019) to infer developmental trajectories of the three MDSC cell types in an unbiased manner, E-, PMN-, and M-MDSC. To order the cells according to pseudotime, the cluster with the highest StemID2 scores was chosen as the root node. StemID2 is included in the RaceID3 package (v0.3.8) and it was used to calculate the nodes connectivity and the transcriptome entropy of each unsupervised cell cluster, which reflects the uniformity of the transcriptome (Rosales-Alvarez et al. 2023). These two parameters were used to calculate the StemID2 scores. The cluster with larger score was considered the origin. The cell origins were validated using gene markers such as *DEFA4* and *PRTN3*, which are well known markers of immature myeloid progenitor cells which originates early myeloid-derived suppressor cells (E-MDSCs) (Basingab et al. 2022; Karatepe et al. 2018).

#### Cell-to-cell interaction analysis

Intercellular interactions were inferred using CellChat R package (v2.1.2) following its suggested workflow and using standard parameters (Jin et al. 2021, 2025). The cell- cell communication analysis was inferred based on the expression of ligand-receptor pairs from the CellChatDB (Jin et al. 2021) using all three subset types: secreted signaling, cell–cell contact and ECM–Receptor.

## Results

We enrolled seven preterm (gestational age ≥ 26 to ≤ 36 weeks) and six full-term (≥ 37 weeks gestational age) neonates. Preterm newborns exhibited clinical characteristics typical of their gestational age (Table 1). None of the preterm neonates showed evidence of infection at the time of blood collection and all met the predefined inclusion and exclusion criteria. Despite the absence of active infection, prenatal histories revealed maternal conditions commonly associated with preterm birth, including preeclampsia, preterm premature rupture of membranes, autoimmune disease, and chorioamnionitis. The majority of mothers of preterm neonates belonged to underrepresented racial groups, consistent with national epidemiological trends (Barreto et al. 2024). The median gestational age and range cohort are provided in Table 1. All preterm neonates were discharged in stable condition at corrected gestational age of > 37 weeks.

### Single cell analysis of PBMCs from preterm neonates reveals significant enrichment of myeloid-derived suppressor cells (MDSC)

**Fig. 1:**
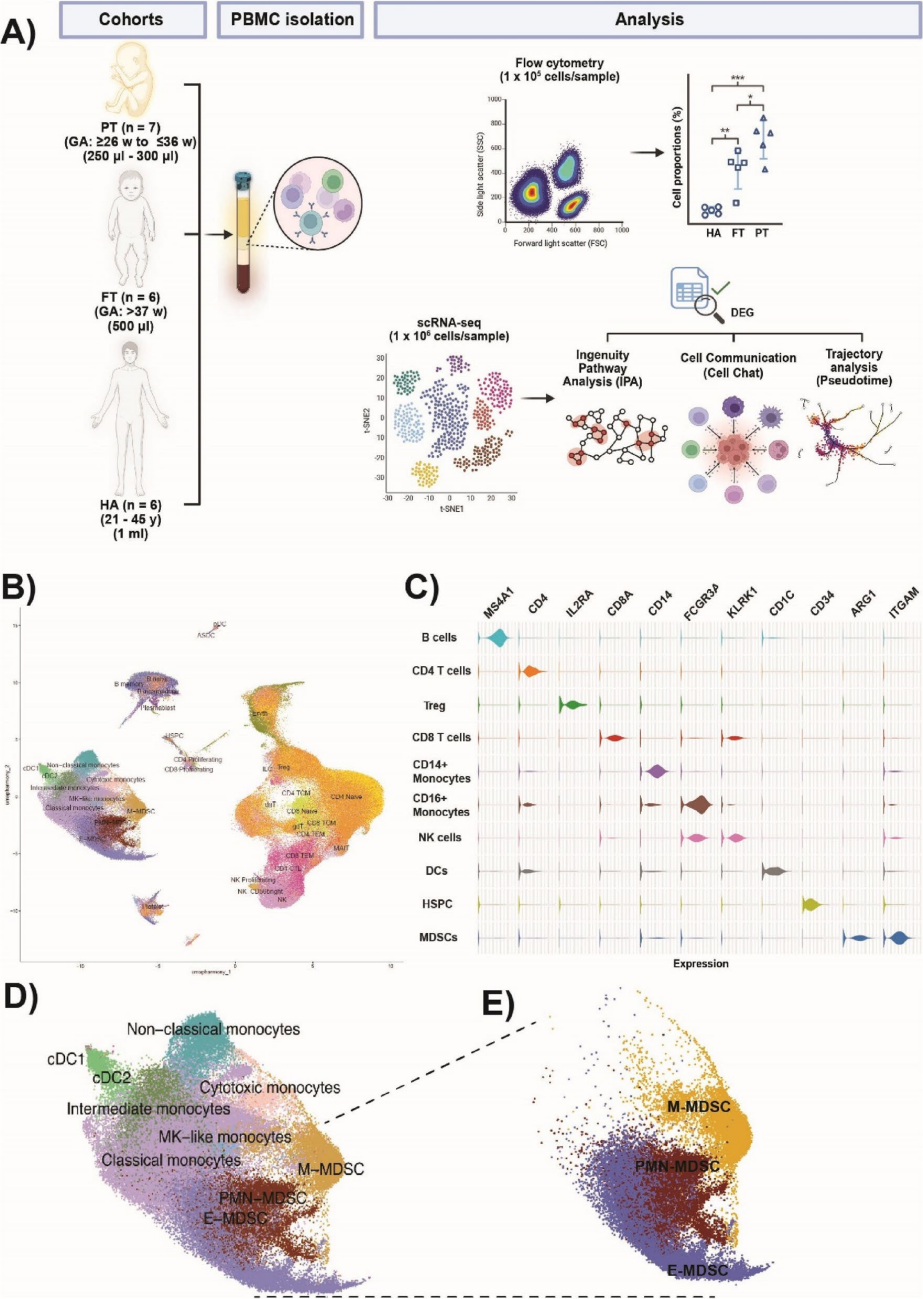
Study overview and immune cell profiling across age groups. **A** Schematic representation of the study design (created with BioRender.com). Peripheral blood samples were collected from three participant cohorts: preterm neonates (PT; gestational age [GA] ≥ 26 to ≤ 36 weeks; *n* = 7), full-term neonates (FT; GA > 37 weeks; *n* = 6), and healthy adults (HA; 21–45 years old; *n* = 6). PBMCs were isolated and analyzed using single-cell RNA sequencing (scRNA-seq). Differentially expressed genes (DEGs) were identified, followed by pathway enrichment analysis, pseudotime analysis, and cell–cell communication inference. Single cell annotation proportions of MDSCs are validated with flow cytometry. **B** UMAP projection of scRNA-seq data from 19 samples, depicting 36 transcriptionally defined immune cell clusters annotated by major cell types. **C** Violin plots of canonical marker gene expression across major immune effector populations, confirming accurate cell-type classification. **D** UMAP projection of myeloid populations, highlighting clusters including monocytic (M-MDSCs), polymorphonuclear (PMN-MDSCs), and early stage MDSCs (E-MDSCs) myeloid-derived suppressor cells (dotted circle). Cluster identities are based on canonical marker expression and transcriptional similarity. **E** UMAP highlighting the three MDSC subsets: M-MDSCs (yellow), PMN-MDSCs (brown), and E-MDSCs (blue)

To generate single-cell RNA-sequencing profiles of PBMCs from preterm and full-term neonates, blood samples were collected during routine clinical draws, either during NICU hospitalization (preterm) or prior to hospital discharge (full-term). Healthy adult participants were included as a reference group (Fig. 1A). Despite reduced blood volumes in preterm (250–300 µl) and full-term (500 µl) neonates, sample viability and cell yield were high (> 90% and ~ 1.0–1.5 × 10^6^ cells per sample). Following quality control filtering, we obtained transcriptomic data on approximately 339,251 cells across all 19 individuals (seven preterm, six full-term, six healthy adults). Unsupervised clustering and analysis based on canonical marker gene expression identified 36 different cell populations within the dataset (Fig. 1B-C).

**Fig. 2:**
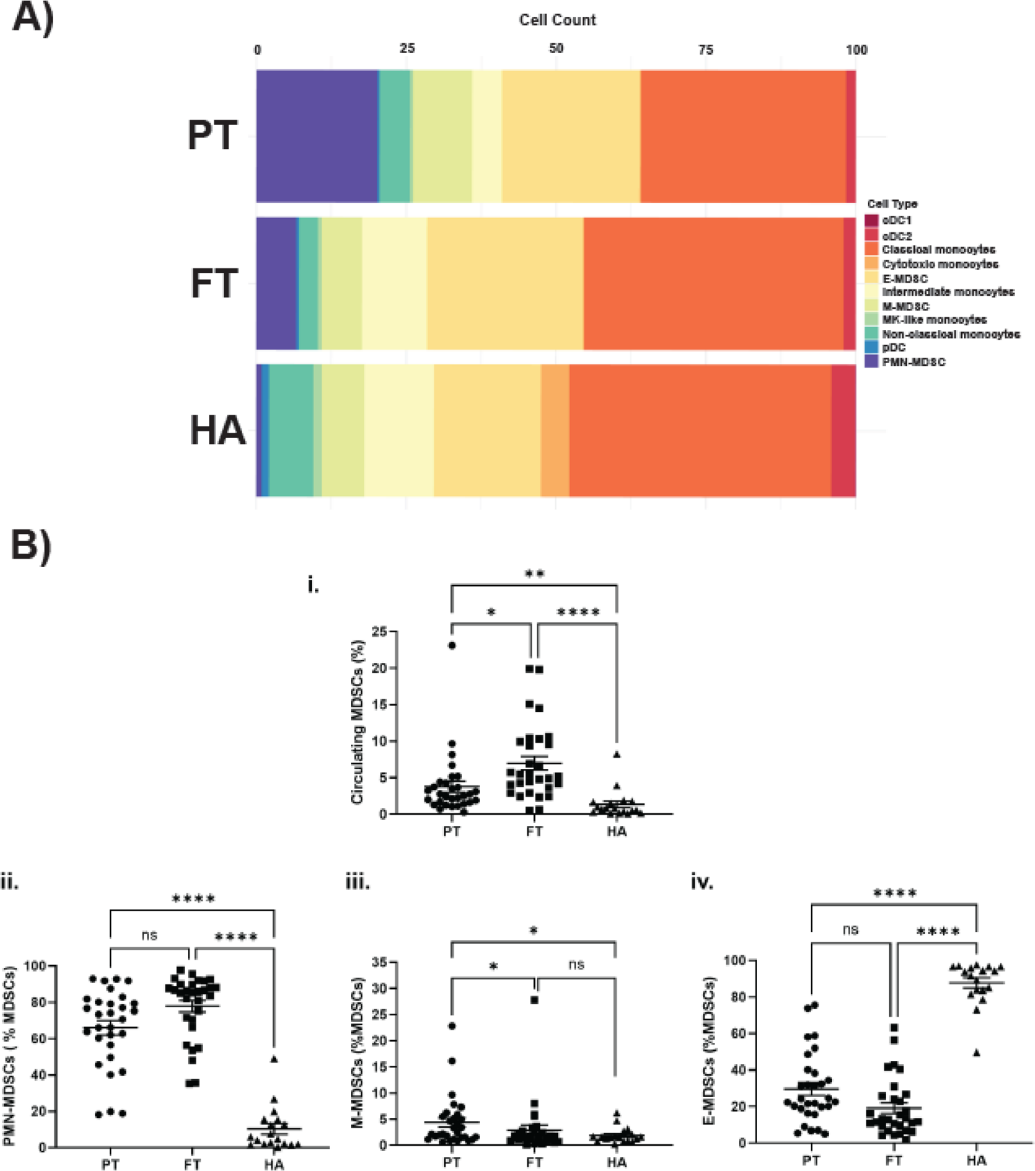
Composition and transcriptional landscape of the myeloid compartment across developmental groups. **A** Stacked bar plot showing the average relative abundance of annotated myeloid cell subsets across preterm (PT) neonates, full-term (FT) neonates, and healthy adult (HA) samples. Each bar represents the mean proportion of each cell type within the total myeloid compartment per group. Notable differences in the prevalence of MDSC subsets, particularly PMN-MDSCs, are observed between preterm (10.56 ± 5.27) and full-term (2.56 ± 1.3) neonates, compared to healthy adults (0.38 ± 1.3), suggesting age-dependent dynamics in myeloid composition. **B** Boxplots showing the distribution of the relative frequency of MDSCs across individual samples per group (PT, FT, HA), as determined by flow cytometry in larger cohorts (PT, *n* = 30; FT, *n* = 30; HA, *n* = 18). Each panel depicts the proportion of (i) total MDSCs (CD11b^+^CD33^+^HLADR^low/−^), (ii) PMN-MDSCs (CD11b^+^CD33^+^HLA-DR^low/−^CD66b^+^), (iii) M-MDSCs (CD11b^+^CD33^+^HLA-DR^low/−^CD14^+^), and (iv) E-MDSCs (CD11b^+^CD33^+^HLA-DR^low/−^CD66b^−^CD14.^−^). Statistical significance was assessed using the Kruskal–Wallis test, followed by Dunn’s multiple comparisons test with Bonferroni correction. ns = not significant, *p < 0.05, **p < 0.01, ***p < 0.001)

**Fig. 3:**
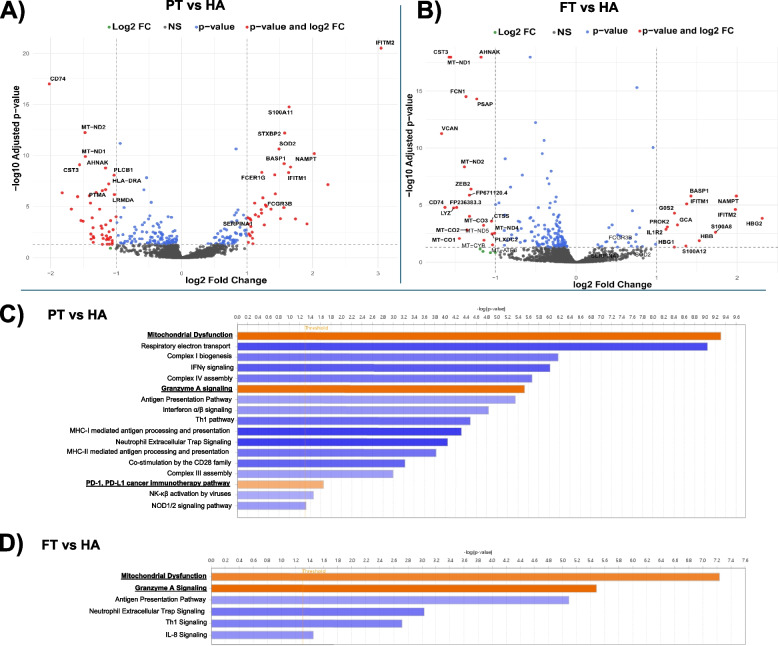
Transcriptional profiling of PMN-MDSCs reveals age-specific gene signatures and pathway enrichment. **A** Volcano plot showing differentially genes (DEGs) in PMN-MDSCs from preterm (PT) neonates compared to healthy adults (HA) and** B** full-term (FT) neonates versus healthy adults. The *apeglm* method from DESeq2 was applied to moderate effect size estimates for low-count or high-variance genes. Red dots represent significantly upregulated genes (adjusted *p*-value < 0.05, log_2_ FC > 1); blue dots indicate significantly upregulated genes that did not meet log_2_FC threshold; gray dots represent non-significant genes. **C-D** Canonical pathway enrichment based on DEGs in PMN-MDSCs from preterm (**C**) and full-term (**D**) neonates relative to healthy adults. Significantly enriched pathways include mitochondrial dysfunction, granzyme A signaling, PD-1/PD-L1 cancer immunotherapy signaling, and antigen presentation. Bar color indicates activation z-scores: orange (z > 2, predicted activation), blue (z < −2, predicted inhibition). Enrichment threshold: –log_10_ (*p*-value) ≥ 1.3, corresponding to *p* < 0.05. A full list of genes associated with each upregulated pathway is provided in Supplementary Material 2

Next, we focused on immune cell populations within the myeloid compartment. Using a semi-supervised clustering analysis, we identified ten distinct cell clusters. Cell identities were assigned based on consensus across multiple reference-based annotation tools to ensure robust classification. This was further refined by assessing cluster-specific expression of canonical myeloid gene markers, enabling more precise subset delineation (see *Methods 2.3.4*). In total, seven well-defined myeloid clusters were identified: classical monocytes, non-classical monocytes, intermediate monocytes, conventional dendritic cells (DC), plasmacytoid DCs, cytotoxic monocytes and megakaryocyte (MK)-like monocytes (Fig. 1D). Three additional clusters showed reduced expression of canonical monocyte markers (e.g., *CD14, FCGR3A, CCR2*) and DC markers (e.g., *HLA* genes). Rather than annotating these clusters by exclusion of known lineages, we applied a semi-supervised approach integrating SingleR and Azimuth results with the expression patterns of established MDSC marker genes. Based on this integrated analysis, these clusters were annotated as MDSCs Molecular signature analysis further supported this classification, revealing high expression of S100 alarmins (*S100A8, S100A9, S100A12*) and other well-characterized MDSC markers including *ARG1 (*Ma et al. 2020), *LTF (*He et al. 2018*), and CD177 *(Yao et al. 2024; Pelosi et al. 2021). Based on canonical gene expression, MDSCs were further subtyped into: Early-stage MDSCs (E-MDSCs; *IL7R*, *CLR1, CEACAM8*), granulocytic MDSCs (PMN-MDSCs; *ARG1*, *HP*, *CD177*, *FCGR3B, MCL1*) and monocytic MDSCs (M-MDSCs; *RABGAP1L*, *NOTCH2*, *SLC22A15*) (Fig. 1E and Fig. S2). Proportional analysis showed that PMN-MDSCs were significantly expanded in preterm neonates (10.56 ± 5.27%) compared to full-term neonates (2.56 ± 1.30%) and healthy adults (0.38 ± 1.30%) (Fig. 2A). These findings were validated by flow cytometry, which also demonstrated increased PMN-MDSC numbers in both preterm and full-term neonates compared to adults (Fig. 2B). Furthermore, at a single-cell level, the relative proportions of all other myeloid subsets (excluding MDSCs) were reduced in neonates compared to adults (Table S2). Differential expression analysis revealed that PMN-MDSCs from both preterm and full-term neonates showed upregulation of *IFTIT1, IFIT2, BASP1, NAMPT,* and *S100A family genes* compared to adults*.* Additional genes selectively upregulated in preterm neonates were *FCER1G*, *FCGR3B*, *SERPINA1*, *SOD2*, *STXBP2* while *G0S2*, *GCA*, *HBB*, *HBG1*, *HBG2*, *IL1R2*, and *PROK2* gene expression was upregulated in full-term neonates (Fig. 3A-B). Subsequent pathway enrichment analysis indicated that neonatal PMN-MDSCs preferentially upregulated genes involved in mitochondrial dysfunction, granzyme A signaling, and the PD-1/PD-L1 checkpoint pathway compared to adults (Fig. 3C-D) These results demonstrate enrichment of canonical MDSC-associated pathways, as previously described in the adult cancer literature (Li et al. 2026), thereby supporting the annotation of neonatal MDSCs using limited-volume (250–500 µL) single-cell transcriptomic profiling. Unexpectedly, differential expression analysis of PMN-MDSCs between preterm and full-term neonates revealed minimal transcriptional divergence, with few genes meeting statistical significance and no enriched pathways identified. Similarly, M-MDSC expression analysis between preterm and full-term neonates only demonstrated three DEGs in M-MDSCs and five DEGs in E-MDSCs, underscoring the substantial transcriptomic similarity between neonatal cohorts. In contrast, comparisons of preterm and full-term neonates to adults revealed consistent downregulation of inflammatory pathways in E- and M-MDSCs, including other multiple canonical pathways identified by IPA (Fig. S3), supporting a conserved neonatal-specific transcriptional program.

### Trajectory analysis reveals unique MDSC maturation pathways in preterm neonates

To investigate developmental relationships among MDSC subsets and determine whether their differentiation is influenced by gestational age and associated prenatal exposures, we performed an unbiased pseudotime trajectory analysis using the integrated single-cell RNA-sequencing dataset. MDSC differentiation potential was first evaluated using the RaceID3/StemID2 algorithm, which infers lineage hierarchies and predicts progenitor-like clusters (Herman & Sagar, 2018). This analysis identified cluster 11 as having the highest StemID2 score, suggesting it contains multipotent progenitor-like cells (Fig S4A).

**Fig. 4:**
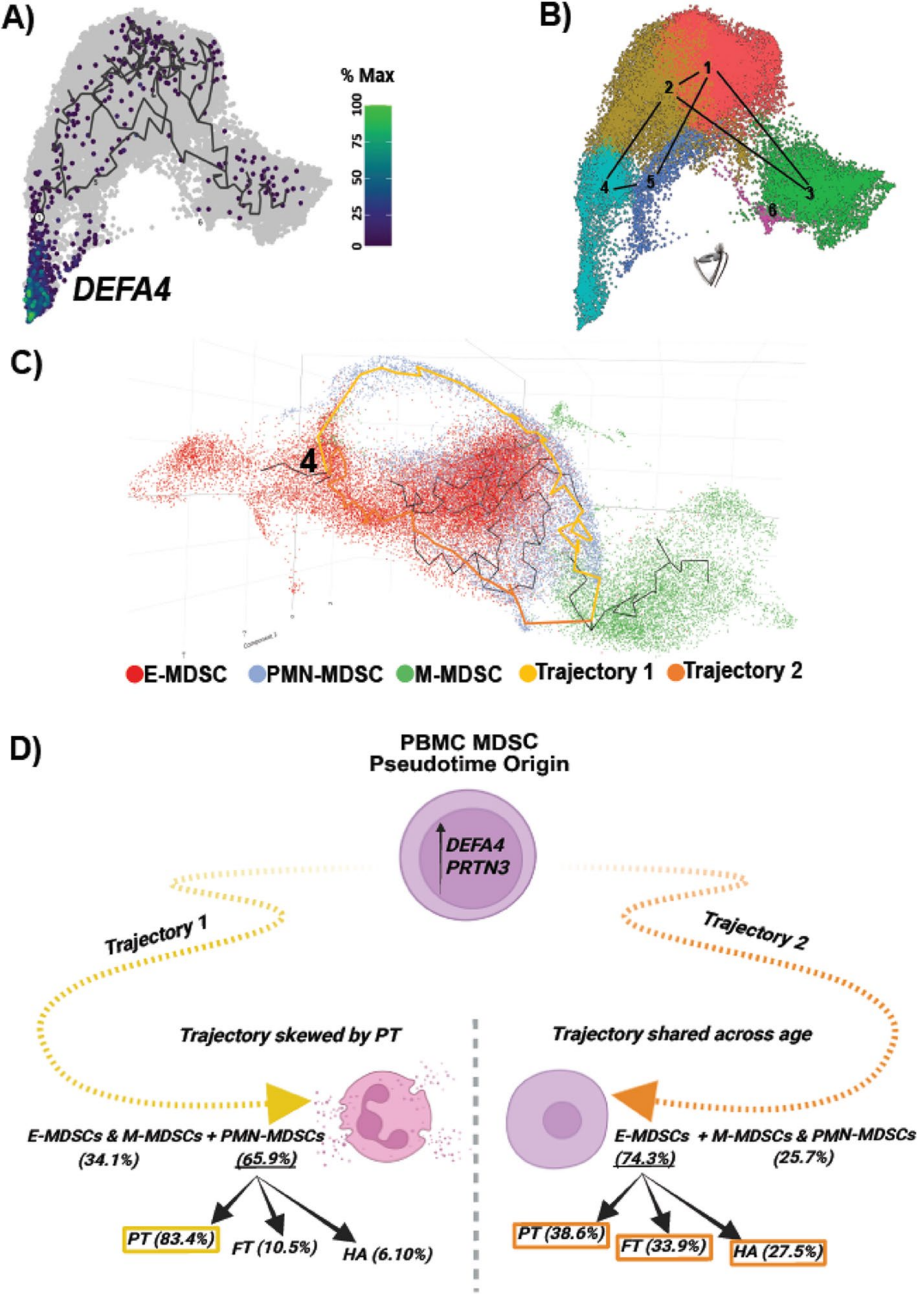
Pseudotime analysis of MDSCs reveals age-associated divergence in developmental pathways. **A–B** Expression of early MDSC marker *DEFA4* confirms the localization of early-stage MDSCs near the trajectory root. **A** UMAP cluster of MDSC subpopulations, represented by expression of *DEFA4*. The black lines represent the pseudotime trajectory. **B** Then, black lines are simplified to interconnections between labeled unsupervised clusters (1–4), *DEFA4* is expressed mostly in cluster 4. The eye symbol is meant to change the reader’s point of view, revealing two distinct trajectories in the following panel. Access Supplementary Material 3 for better visualization. **C** UMAP projection of pseudotime analysis, including a three-dimensional trajectory reconstruction colored by cell type and two distinct trajectories. **D** Trajectory 1 (Tr1) predominantly leads to PMN-MDSC generation (65.9%), was enriched in PT neonates (83.4% compared to 10.5% in FT and 6.1% in HA). Trajectory 2 (Tr2) involves mainly E-MDSCs (74.3%), which share similar proportions across the three cohorts studied (PT: 38.6%, FT: 33.9%, HA: 27.5%). See also Fig. S4 for further details regarding identification of pseudotime origin

**Fig. 5:**
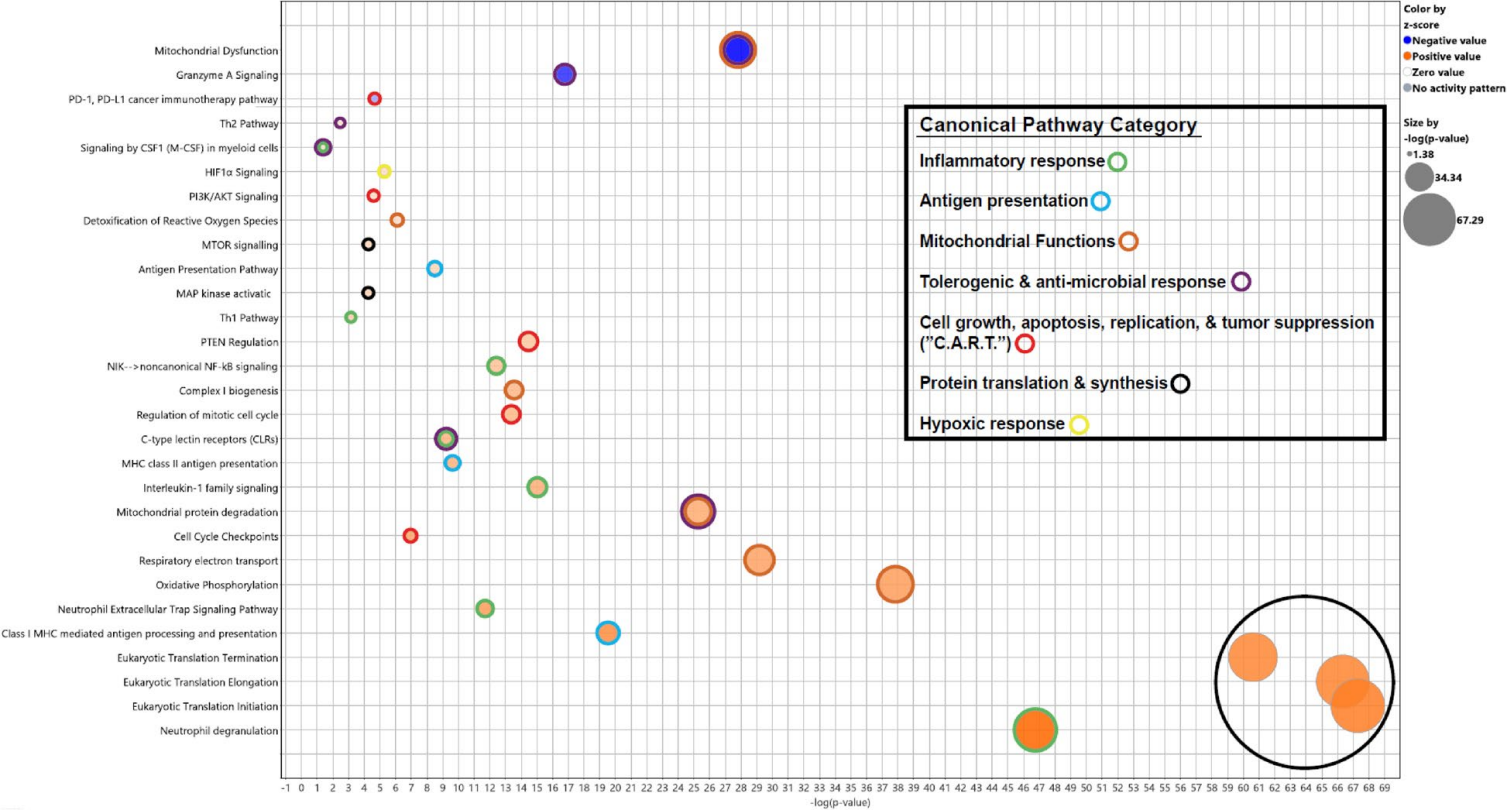
Functional gene analysis of Tr1 vs Tr2 PMN-MDSCs which upregulate the inflammatory response, protein translation and synthesis, “C.A.R.T.”, antigen presentation, and mitochondrial functions and stress response. They also upregulate pathways related to tolerogenic functions (i.e., production of nitric oxide and reactive oxygen species in addition to their detoxification) and downregulate anti-microbial functions (Granzyme A signaling)

Interestingly, this cluster marked the origin of the main pseudotime trajectory, composed largely of E-MDSCs, as confirmed by the expression of early myeloid markers *DEFA4* and *PRTN3* (Fig. 4A-C and S4B-C). Trajectory analysis using Monocle3 further identified cluster 4 as the origin of the inferred lineage tree (Fig. 4A-C). Three-dimensional pseudotime visualization of MDSCs across all age groups revealed two dominant developmental branches (Fig. 4C-D and Supplementary Material 3). Trajectory 1 (Tr1) was predominantly composed of preterm-derived cells (4,894; 83.37%), with fewer cells from full-term neonates (617; 10.51%) and healthy adults (359; 6.12%). In contrast, trajectory 2 (Tr2) exhibited a more balanced distribution: 1,764 (38.60%) cells from preterm neonates, 1,550 (33.92%) cells from full-term neonates, and 1,255 (27.47%) cells from healthy adults. These findings suggest an age-associated divergence in MDSC developmental pathways: Tr1 predominantly represents preterm-associated PMN-MDSC differentiation, while Tr2 reflects a more canonical pathway resulting in E-MDSCs more uniformly across all age groups. Pathway enrichment analysis of Tr1-associated PMN-MDSCs (Fig. 5) revealed upregulation of gene programs related to inflammatory responses, protein translation and biosynthesis, cell growth, apoptosis, replication, tumor suppression, antigen presentation, mitochondrial functions and stress response, as well as tolerogenic and antimicrobial activity. Conversely, Tr1 cells showed downregulation of canonical pathways including mitochondrial dysfunction, Granzyme A signaling, and PD-1-PD-L1 checkpoint signaling relative to Tr2 within the trajectory framework. This complementary analysis of MDSC differentiation revealed that Tr1 represents a distinct, preterm-enriched differentiation trajectory potentially leading to a transcriptionally unique PMN-MDSC subset, characterized by relative attenuation of these canonical pathways compared to Tr2, without contradicting the neonatal-versus-adult enrichment described in Sect. 3.1. In contrast, Tr2 reflects a more conserved developmental program consistent with the cross-sectional findings in Sect. 3.1. and with the existing literature, giving rise to immunosuppressive and protective MDSCs more evenly distributed across gestational and postnatal age.

### Intercellular communication between MDSCs and immune effector cells is age specific

**Fig. 6:**
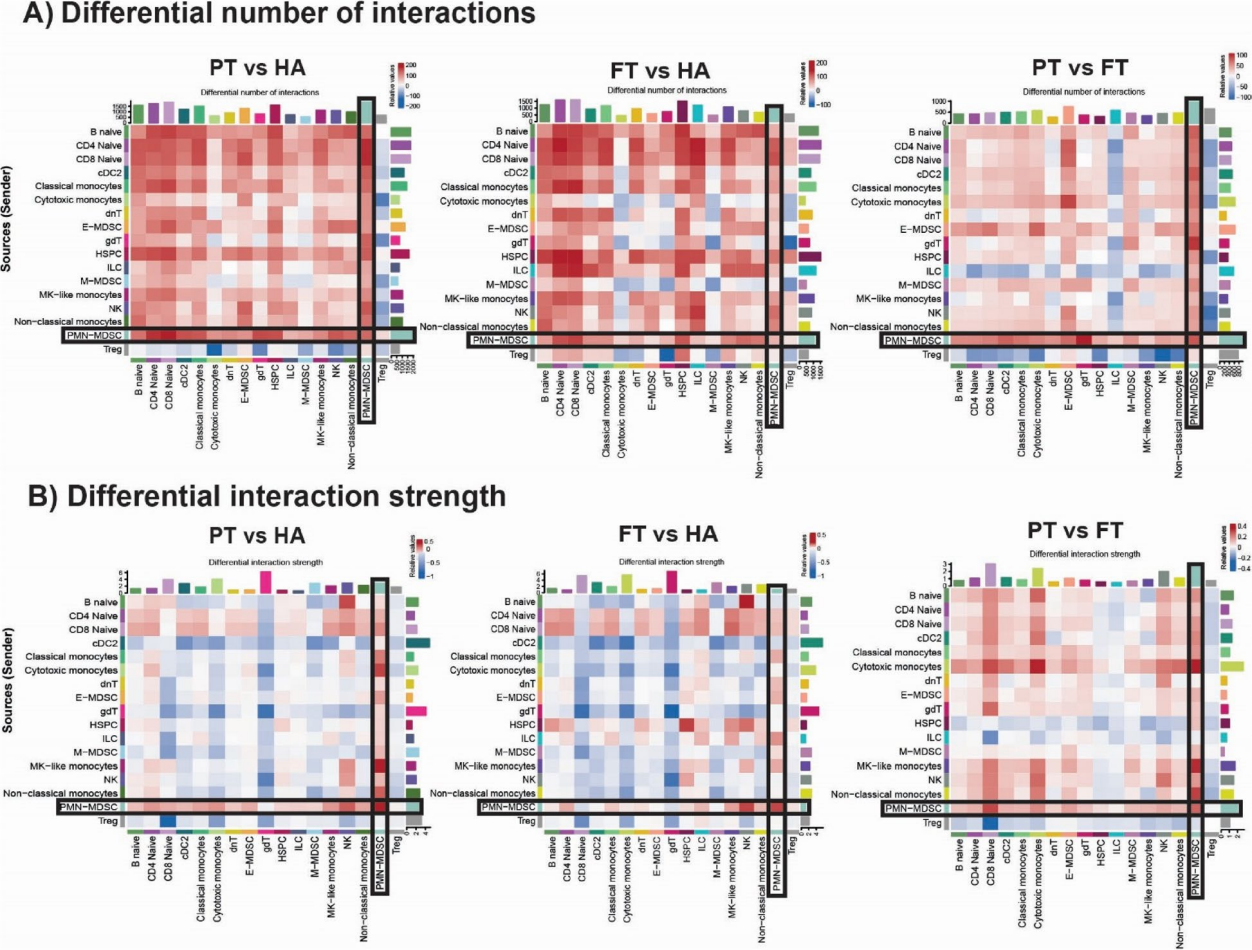
Intercellular communication between MDSCs and immune effector cells is age specific. **A** Differential number of cell–cell interactions based on ligand-receptor pathways from source (sender; Y-axis) to target (receiver; X-axis) cells in preterm (PT) and full-term (FT) neonates compared to healthy adults (HA). **B** Differential interaction strength between sender and receiver cells, based on ligand-receptor signaling, across groups. Red indicates upregulation and blue indicates downregulation. Consistent with Fig. S5, neonates exhibit a higher number of total interactions than healthy adults. However, the interaction strength in healthy adults is greater overall. Regarding PMN-MDSCs, preterm-derived PMN-MDSCs exhibit more frequent and stronger interactions with CD8^+^ T cells, CD4^+^ T cells, and NK cells, as well as robust autocrine signaling. Conversely, regulatory T cells (Tregs) display a progressive decrease increase in both the number and strength of interactions with decreased, suggesting increased myeloid-derived regulation of surrounding PBMCs

Unlike MDSCs isolated from cancer patients, widely recognized for their immunosuppressive functions, MDSCs in preterm neonates have been reported to exhibit reduced immunosuppressive and antimicrobial capacity (Yao et al. 2024). To assess how MDSCs interact with other circulating immune cells during early life, we analyzed ligand-receptor signaling networks using CellChat, a computational framework that infers intercellular communications based on known ligand-receptor interactions (Jin et al. 2025). This analysis predicts both the major incoming and outgoing signals of each cell type and how these interactions contribute to coordinated immune responses. Our results showed that MDSCs from both preterm and full-term neonates exhibited altered interaction networks compared to adults, particularly within the myeloid compartment (Fig. S5). Notably, neonatal PMN-MDSCs exhibited a higher frequency of predicted interactions with multiple immune cell populations compared to adult-derived MDSCs. These included adaptive immune cells (naive CD4^+^ and CD8^+^ T cells), innate immune cells (DCs, monocytes, γδT cells, and NK cells), as well as hematopoietic stem progenitor cells (HSPCs) (Fig. 6A). Additionally, PMN-MDSCs from pre-term neonates exhibited significantly stronger overall interaction strength compared to those from full-term neonates and adults, especially with NK cells, naïve CD4^+^ T cells, naïve CD8^+^ T cells, and other PMN-MDSCs (autocrine signaling) (Fig. 6B). These results suggest that MDSC-mediated immune crosstalk is developmentally regulated, and that preterm MDSCs possess a distinct communication profile, potentially reflective of compensatory mechanisms or altered maturation dynamics in early extrauterine life. The findings further suggest that preterm neonatal immune regulation is driven by myeloid-derived regulators (PMN-MDSCs) in the immediate perinatal period, instead of signaling from Tregs (which predominates more in adults and more moderately in full-term neonates).

### PMN-MDSC from preterm neonates prioritize unique signaling networks

The enhanced outgoing and incoming signaling activity observed in neonatal PMN-MDSCs suggests a substantial impact in modulating immune effector cell communication through several key networks, including ADGRE, RESISTIN, TGFβ, ANNEXIN, SELPLG, ICAM, PECAM1, VISFATIN, and IL4, many of which were diminished or absent in adults (Fig. S6). These cells also exhibited a greater cumulative information flow across all PBMCs by age group, highlighting their central role in shaping immune signaling networks (gray bars, Fig. S6). The relevance of these communication networks is further supported by both total and relative information flow profiles across age groups (Fig. S7). Several networks, including RESISTIN, ADGRE, ANNEXIN, SELPLG, ICAM, VISFATIN, PECAM1, TGFβ, and IL4, demonstrated increased information flow in preterm compared to full-term neonates (Fig. S7A). Further analysis revealed age-specific dominance of individual networks, suggesting developmental regulation of immune communication (Fig. S7B).

**Fig. 7:**
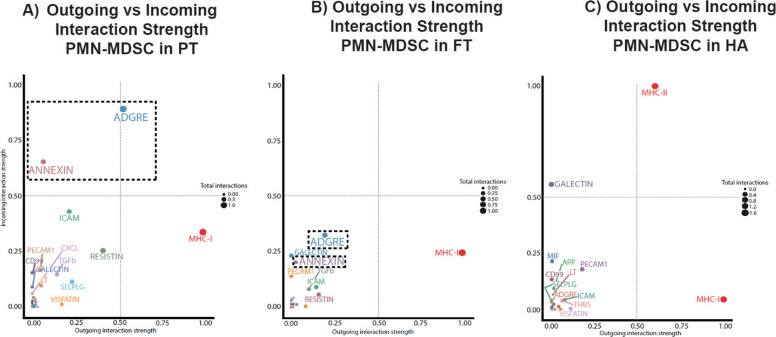
Inferred signaling activity of PMN-MDSCs across developmental groups. Scatter plots showing outgoing versus incoming signaling strength for PMN-MDSCs in preterm (PT), full-term (FT), and healthy adult (HA) samples. Each dot represents a ligand-receptor signaling pathway involving PMN-MDSCs; dot size reflects the total interaction magnitude (incoming + outgoing). Preterm-derived PMN-MDSCs show greater signaling diversity and strength, with prominent incoming signals through pathways such as ADGRE, ANNEXIN, ICAM, RESISTIN, VISFATIN, and TGFβ. In contrast, healthy adult-derived PMN-MDSCs show reduced interaction diversity but exhibit strong incoming signals via MHC-II signaling. Preterm-derived PMN-MDSCs, however, exhibit enhanced regulation of endogenous antigens via incoming MHC-I signals. The dashed square highlights the most notable pathway shifts in preterm samples

To better characterize the directional dynamics of intercellular communication, we compared outgoing and incoming signaling strengths in PMN-MDSCs across age groups. This analysis revealed age-dependent variations. For example, ADGRE exhibited strong bidirectional signaling, while ANNEXIN, RESISTIN, and ICAM were predominantly incoming, and VISFATIN, SELPLG, and TGFβ were largely outgoing in preterm neonates (Fig. 7).

Interestingly, although endogenous antigen presentation via MHC-I signaling was preserved across all age groups (as indicated by strong outgoing MHC-I signals), PMN-MDSCs from preterm neonates showed increased MHC-I incoming signals, suggesting heightened antigen uptake or processing. In contrast, MHC-II interactions were negligible or absent in both preterm and full-term neonates, indicating age-specific impairment or lack of prioritizing exogenous antigen presentation by PMN-MDSCs. Furthermore, GALECTIN incoming signaling was more prominent in adult PMN-MDSCs from adults showed more incoming GALECTIN signaling, whereas preterm and full-term neonates displayed more comparable levels. Notably, network outliers among preterm neonates demonstrated markedly increased interaction strength in several pathways, including ADGRE, ANNEXIN, ICAM, RESISTIN, VISFATIN, TGFβ, and SELPLG. These findings suggest that the PBMC compartment in preterm neonates, shaped by the pro-inflammatory pathophysiology of fetal development and premature birth (Polcz et al. 2023; Yang et al. 2019; Ghaebi et al. 2017; Deshmukh and Way 2019), exhibits heightened signaling activity in PMN-MDSC-associated pathways. This may contribute to a maladaptive immune response and an increased risk of inflammation-induced organ injury.

## Discussion

Myeloid-derived suppressor cell (MDSC) function and transcriptomic profiles have been extensively studied in adults, particularly in the contexts of sepsis and cancer (Hegde et al. 2021). However, their ontogeny and expansion during early life remain poorly understood, despite their potential relevance in shaping neonatal immune responses. In this study, we addressed this knowledge gap by characterizing circulating MDSCs across age-stratified neonatal cohorts using single-cell RNA sequencing and cell-to-cell communication analyses. Specifically, we investigated how prematurity influences MDSC function, differentiation, and signaling. While previous work has characterized neonatal MDSCs (Yao, 2024), our study adds valuable insight by providing a comprehensive comparison of all MDSC subpopulations and their gene expression profiles relative to other PBMC subsets. Importantly, our pseudotime trajectory analysis incorporates both M-MDSC and PMN-MDSC subsets, highlighting a distinct developmental trajectory and biological relevance of PMN-MDSCs in the context of prematurity. Furthermore, our CellChat analysis provides a system-wide view of ligand-receptor interactions across the full PBMC landscape, providing functional insights into MDSC-mediated signaling that would otherwise be difficult to obtain given the limited blood volumes in neonates.

Analysis of the myeloid and lymphoid compartments revealed striking age-dependent differences in both cellular composition and subset abundance. Immune development begins in utero, and postnatal transitions are particularly pronounced during early life (Kalagiri et al. 2016; Köstlin-Gille and Gille 2020). Single-cell profiling of PBMCs from preterm neonates demonstrated significant enrichment of MDSCs, particularly PMN-MDSCs, relative to full-term neonates and adults as previously documented (Yao, 2024; Köstlin-Gille and Gille 2020). This disproportionately high representation of PMN-MDSCs in preterm neonates reflects a distinct early-life myeloid signature. In contrast, the myeloid compartment in adults was dominated by classical and intermediate monocytes, indicative of a more mature and functionally specialized innate effector cells primed for immune homeostasis. Flow cytometry confirmed this age-related shift, supporting earlier reports that MDSC frequencies decline after birth (Köstlin-Gille and Gille 2020). The observed expansion of PMN-MDSCs in preterm neonates may reflect ongoing immune development (Pronovost and Hsiao 2019), or a response to perinatal inflammation (Kamdar et al. 2020), underscoring the influence of developmental context on MDSC biology.

Previous studies have shown that neonatal MDSCs contribute to immunosuppression and Th2 polarization (Köstlin-Gille and Gille 2020; Wegmann et al. 1993). These Th2-skewed responses have been reported in single-cell analysis of cord blood (Köstlin-Gille and Gille 2020), whole blood (Olaloye et al. 2025) and PBMCs (He et al. 2018; Yao, 2024). Additionally, MDSCs are known to promote Th2 immunity in pediatric asthma (Brauner et al. 2025). Consistent with this, we observed that PMN-MDSCs from both preterm and full-term neonates expressed genes associated with immune regulation and antimicrobial responses. Notably, they also showed transcriptional signatures linked to immune checkpoint pathways (e.g., PD-1/PD-L1) (Wang JC & Sun L., 2022), mitochondrial dysfunction (Weiss et al. 2020; Ohl and Tenbrock 2018; Xie et al. 2025), and inflammatory mediators (e.g., S100 alarmins) (Brauner et al. 2025; He et al. 2018; Sinha et al. 2008), previously described in MDSCs from cancer patients. This dual expression pattern suggests that neonatal PMN-MDSCs are transcriptionally poised to suppress immune responses while also participating in inflammatory and metabolic stress signaling. Compared to adults, neonatal PMN-MDSCs showed increased expression of both immunosuppressive and pro-inflammatory genes, suggesting a dual role in shaping early immune environment.

To investigate MDSCs developmental dynamics, we performed pseudotime trajectory analysis and identified two distinct transcriptional trajectories. Trajectory 1 (Tr1), enriched in preterm neonates, led primarily to PMN-MDSC differentiation characterized by gene programs involved in pro-inflammatory signaling, protein translation, mitochondrial stress pathways (mTOR, AMPK, HIF1α) and tolerogenic pathways. In contrast, trajectory 2 (Tr2), which was more evenly represented across all age groups, generated E-MDSCs with transcriptional signatures associated with classical immune regulation and antimicrobial activity. These findings suggest that prematurity promotes an alternative MDSC maturation pathway (Tr1), skewing immune responses toward inflammation and stress, which may impair the balance between tolerance and host defense and increase susceptibility to immune dysregulation.

To further explore the role of MDSCs in neonatal immunity, we examined predicted ligand-receptor signaling. PMN-MDSCs from preterm neonates demonstrated a significant higher number and strength of interactions with both innate and adaptive immune cells, particularly naive CD8^+^ and CD4^+^ T cells, and increased autocrine signaling. These findings suggest a heightened potential for immune modulation in preterm neonates. Network analysis revealed enhanced signaling via pathways including ADGRE, RESISTIN, TGFB, ANNEXIN, SELPLG, ICAM, PECAM1 VISFATIN, and IL-4, many of which were diminished or absent in adults. Directional analysis showed that some pathways, such as ADGRE, exhibited bidirectional signaling, ANNEXIN exhibited more incoming signals, while others, like VISFATIN, SELPLG, and TGFB, were largely outgoing in preterm PMN-MDSCs. These pathways, largely diminished or absent in adults, suggest a distinct intercellular communication profile that may contribute to altered immune regulation in preterm neonates. The changes in ADGRE and ANNEXIN networks in PMN-MDSCs from preterm neonates most likely reflect the robust state (Kamdar et al. 2020) of cell surface molecules involved in cell signaling due to the organism’s proinflammatory gestation and recent birth (Yang et al. 2019; Ghaebi et al. 2017; Deshmukh and Way 2019).

Antigen presentation also showed age-related differences. While MHC-I PMN-MDSC outgoing signaling was preserved, MHC-II interactions were reduced or absent in MDSCs from neonates. However, preterm neonates exhibited greater MHC-I incoming signals to PMN-MDSCs from other PBMCs. This could indicate that preterm development and birth skews PMN-MDSCs to process more endogenous than exogenous antigens, possibly contributing to development of hypersensitivity reactions and auto-immune diseases (Brauner et al. 2025; McGonagle et al. 2009; Protić-Rosić et al. 2025). Additionally, GALECTIN signaling, more prominent in adult PMN-MDSCs, was comparable in preterm and full-term neonates but showed significant heterogeneity on the preterm cohort. These differences underscore a unique pattern of signaling networks associated with antigen processing and extracellular matrix proteins in preterm PMN-MDSCs, shaped by the inflammatory pathophysiology of premature gestation and birth.

In summary, our analysis reveals preterm neonates exhibit disproportionate expansion of PMN-MDSCs that follow a distinct differentiation trajectory and exhibit enhanced signaling with specific immune cells. While classical Tr2 appeared conserved across ages and supported immunotolerance, the alternative Tr1 differentiation observed in preterm neonates (Tr1) reflects a shift toward pro-inflammatory and dysfunctional tolerogenic programs that may compromise effective immune regulation. This divergence may contribute to the increased vulnerability of preterm neonates to infection, inflammation, and impaired immune maturation. These findings have important implications for neonatal immunology and immune-targeted therapies. If pathological PMN-MDSC programming drives inappropriate inflammation or impaired tolerance, modulating differentiation to favor a Tr2-like profile could restore immune homeostasis.

## Conclusions

In this single-center cohort, prematurity was associated with a distinct developmental trajectory of MDSCs, driven by expansion of PMN-MDSCs that exhibited pro-inflammatory and metabolically stressed transcriptional programs. These transcriptional programs deviate from the immunotolerant, homeostatic trajectories observed in full-term neonates and adults, suggesting that premature birth disrupts myeloid regulating cells during gestation, transition to, and immediately after extrauterine life.

Beyond defining developmental divergence, our findings position PMN-MDSCs as key regulators of early neonatal immunity. Enhanced intercellular signaling, particularly through ADGRE, RESISTIN, TGF-β, ANNEXIN, and ICAM networks, points to a heightened yet dysregulated communicative capacity that may exacerbate inflammation or impede coordinated immune resolution. The identification of these skewed signaling axes opens new avenues for therapeutic modulation of MDSC differentiation or function to restore immune equilibrium in premature neonates. The plan to facilitate investigations of these new avenues will be to compare the results of this study with PBMC samples of currently enrolled septic preterm neonates.

Taken together, these data bridge mechanistic insight with translational relevance: they implicate pathological MDSC programming as a driver of immune dysfunction and tissue damage in preterm neonates. If validated functionally, strategies that redirect PMN-MDSCs toward canonical, tolerogenic differentiation (whether through metabolic reprogramming, cytokine modulation, or postnatal immunotherapy) may hold promises for reducing infection-related morbidity and improving long-term immune outcomes in this high-risk population.

### Limitations

This study has several limitations. First, the limited blood volume obtainable from preterm neonates restricted downstream validation of transcriptomic findings with functional or metabolic assays. Second, while our cohort captures the critical early neonatal window, the relatively modest sample size, especially among extremely preterm neonates, restricts stratified analyses across narrower gestational ranges. Given the known clinical and biological heterogeneity of preterm birth, including differences in gestational ages and prenatal exposures, the findings should be interpreted as hypothesis-generating rather than definitive. This cross-sectional study was designed to pilot future investigations with larger cohorts and ultimately contribute to the development of a reference transcriptomic framework for disease states such as neonatal sepsis. Furthermore, although the use of healthy adults as a reference group for preterm neonates has inherent limitations, this approach was necessary given the scarcity of published single-cell PBMC datasets in neonates. Adult samples provided well-characterized immune reference points and enabled validation of cluster identity using established canonical transcriptomic pathways. Importantly, conclusions regarding prematurity-associated effects are based on direct comparisons between preterm and full-term neonates, whereas adult samples were used to contextualize neonatal immune signature rather than to define prematurity-specific differences. Third, scRNA-sequencing captures transcriptional states, which do not always reflect protein activity; and ligand-receptor interactions remain predictive. There is a possibility that the identified PMN-MDSC single-cell clusters in neonates may include low-density neutrophils (LDNs) consequently of isolating PBMC compartment and thereby excluding mature granulocytes (high-density neutrophils). In the absence of functional assays, definitive confirmation of suppressive activity of PMN-MDSCs cannot be established nor differentiated them from LDNs. However, since the distinction between PMN-MDSCs and LDNs remains an area of ongoing investigation (Seman and Robinson 2021; Nutley, et al., 2025), particularly outside the context of cancer; this study designated PMN-MDSCs in the context of neonatal immunology based on established phenotypic markers, their presence within PMBC compartment, and enrichment of canonical MDSC associated transcriptional pathways.

Substantial clinical differences between the preterm and full-term cohorts (e.g., cesarean delivery, chorioamnionitis, preeclampsia, gestational diabetes, hypertension, smoking exposure, and antenatal betamethasone administration) may act as confounding or modifying factors influencing immune development during gestation and early postnatal life. While these exposures reflect the clinical context in which preterm birth occurs, they may contribute to inter-individual variability in immune phenotypes. Finally, although our analyses identified expansion of myeloid-derived immune regulatory populations with predominantly pro-inflammatory transcriptional programs, interpretation of these functional states is based on transcriptomic inference and requires further validation.

These limitations are balanced by consistent single-cell transcription patterns across individuals and concordance with flow cytometric estimates of MDSC abundance. Future longitudinal and multicenter studies integrating functional validation will be essential to dissect gestational subgroups and test whether targeting MDSC programs can improve clinically meaningful outcomes in preterm neonates.

## Supplementary Information

Supplementary Material 1.

Supplementary Material 2.

Supplementary Material 3.

## Data Availability

Raw sequencing data have been deposited in the National Center for Biotechnology Information (NCBI) Sequence Reads Archive (SRA) with ascension ID (ACCESSION NUMBER).

## Abbreviations

AMPK: AMP-activated protein kinase
ASDC: AXL⁺ Siglec-6⁺ dendritic cells
BCx: Blood culture
BSA: Bovine serum albumin
cDC1: Conventional dendritic cell type 1
cDC2: Conventional dendritic cell type 2
CTL: Cytotoxic T lymphocyte
DC: Dendritic cell
DEG: Differentially expressed gene(s)
ECM: Extracellular matrix
EDTA: Ethylenediaminetetraacetic acid
E-MDSC: Early myeloid-derived suppressor cell(s)
EOS: Early-onset sepsis
FDR: False discovery rate
FT: Full-term neonate(s)
GA: Gestational age
GBS: Group B Streptococcus
HA: Healthy adult(s)
HIF1α: Hypoxia-inducible factor 1 alpha
HLA: Human leukocyte antigen
HLA-DR: Human leukocyte antigen–DR
HSPC: Hematopoietic stem and progenitor cell(s)
ICAM: Intercellular adhesion molecule
IL: Interleukin
ILC: Innate lymphoid cell(s)
IPA: Ingenuity Pathway Analysis
IRB: Institutional Review Board
IVH: Intraventricular hemorrhage
LOS: Late-onset sepsis
MAIT: Mucosal-associated invariant T cell(s)
MDSC: Myeloid-derived suppressor cell(s)
MHC-I: Major histocompatibility complex class I
MHC-II: Major histocompatibility complex class II
MK: Megakaryocyte
mRNA: Messenger RNA
mTOR: Mechanistic target of rapamycin
NCBI: National Center for Biotechnology Information
NEC: Necrotizing enterocolitis
NICU: Neonatal intensive care unit
NK: Natural killer cell(s)
NO: Nitric oxide
PBMC: Peripheral blood mononuclear cell(s)
PD-1: Programmed cell death protein 1
PD-L1: Programmed death-ligand 1
PMN-MDSC: Polymorphonuclear myeloid-derived suppressor cell(s)
PPROM: Preterm premature rupture of membranes
PT: Preterm neonate(s)
RNA-seq: RNA sequencing
ROS: Reactive oxygen species
scRNA-seq: Single-cell RNA sequencing
SRA: Sequence Read Archive
ssGSEA: Single-sample gene set enrichment analysis
T1: Timepoint 1 (24–48 h after birth)
TGF-β: Transforming growth factor beta
Th2: T helper type 2 cell(s)
Treg(s): Regulatory T cell(s)
UMAP: Uniform Manifold Approximation and Projection
