# Supplementary material for: Preterm birth skews the developmental trajectory of myeloid-derived suppressor cells in the first 48 hours of life: single-cell transcriptomics and inferred intercellular communication
Source: Mol Med. 2026 Mar 27;32:69. doi: 10.1186/s10020-026-01459-8 (PMC13147740; doi:10.1186/s10020-026-01459-8)
Supplement: Supplementary file 1 — Supplementary Material 1. [file 10020_2026_1459_MOESM1_ESM.docx]

**Supplementary Materials:**

- Table S1: Cohort Characteristics of Flow Cytometry Subjects.
- Key Resources: Flow Antibody Table
- Table S2: Total PBMC Count (Proportion %).
- Supplementary Files (Additional Files):
  - Figure 3 Supplementary File (Excel)– Genes associated with canonical pathways
    - Table S5: List of genes associated with upregulation of Mitochondrial Dysfunction in preterm neonates compared to healthy adults
    - Table S6: List of genes associated with upregulation of Mitochondrial Dysfunction in fullterm neonates compared to healthy adults
    - Table S7: List of genes associated with upregulation of Granzyme A Signaling in preterm neonates compared to healthy adults
    - Table S8: List of genes associated with upregulation of Granzyme A Signaling in fullterm neonates compared to healthy adults
    - Table S9: List of genes associated with upregulation of PD-1/PD-L1 cancer immunotherapy pathway in preterm neonates vs healthy adults
  - Figure 4 Supplementary File – Pseudotime Trajectory Video
- Figure S1. Gating strategy for the identification of circulating MDSC subsets.
- Figure S2. Myeloid dot plot.
- Figure S3. Transcriptional profiling of E- and M-MDSCs reveals age-specific gene signatures and pathway enrichment
- Figure S4. Validation of trajectory root location.
- Figure S5. Intercellular communication between MDSCs and immune effector cells is age specific.
- Figure S6. Age-dependent differences in overall signaling activity and PMN-MDSC-specific ligand-receptor communication.
- Figure S7. Comparison of information flow across signaling pathways by age group.

| **Table S1: Cohort Characteristics of Flow Cytometry Subjects** | | | | | | |
| --- | --- | --- | --- | --- | --- | --- |
| **Patient characteristics** | | **Preterm Neonates (n = 32)** | | | **Full Term Neonates (n = 25)** | **Healthy Adults (n = 11)** |
| *Demographics* | |  | | |  |  |
| Median age week gestational age or years (range) | | 31.9, (26 – 34.29) w GA | | | 39.57, (37 – 43) w GA | 29, (25 – 34) years |
| Median birthweight (range) | | 1547.5 (445 – 2940) g | | | 3420 (2730 – 4690) g | N/A |
| Median corrected GA at T1 blood collection, (range) | | 32.14, (26.43 – 34.71) w GA | | | N/A | N/A |
| Median corrected GA at discharge, (range) | | 37.22, (35.14 – 47.29) w GA | | | N/A | N/A |
| Male, n (%) | | 20 (62.5%) | | | 13 (52.0%) | 7 (63.6%) |
| Apgar scores (mean score 1min / 5 min) | | 6 / 8 | | | 8 / 9 | N/A |
|  | *Ethnicity (mean, %)* |  | | |  |  |
|  | Hispanic/Latino | 1 (3.12%) | | | 4 (16.0%) | 1 (9.09%) |
|  | Not Hispanic/Latino | 31 (96.8%) | | | 21 (84.0%) | 10 (90.9%) |
|  | Not reported | 0 | | | 0 | 0 |
|  | *Race (mean, %)* |  | | |  |  |
|  | White/Caucasian | 23 (71.9%) | | | 15 (60.0%) | 10 (90.9%) |
|  | Black/African American | 10 (31.3%) | | | 5 (20.0%) | 0 |
|  | American Indian/Alaskan native | 0 | | | 0 | 0 |
|  | Hawaiian native/Pacific Islander | 0 | | | 0 | 0 |
|  | Asian | 0 | | | 1 (4.0%) | 1 (9.09%) |
|  | Other | 0 | | | 4 (16.0%) | 0 |
|  | Not reported | 0 | | | 0 | 0 |
|  | Maternal prenatal history (mean, %) | |  |  | |  |
|  | Prenatal Care | | 32 (100%) | 24 (96.0%) | | N/A |
|  | Auto-immune disease | 0 | | | 1 (4.0%) | N/A |
|  | Tobacco use | 1 (3.13%) | | | 1 (4.0%) | N/A |
|  | Toxicology screen | 5 (15.6%) | | | 1 (4.0%) | N/A |
|  | Preeclampsia | 20 (62.5%) | | | 7 (25%) | N/A |
|  | Magnesium exposure | 28 (87.5%) | | | 2 (8.0%) | N/A |
|  | Betamethasone exposure | 30 (93.8%) | | | 0 | N/A |
|  | Gestational Diabetes | 3 (9.38%) | | | 4 (16.0%) | N/A |
|  | Chorioamnionitis | 4 (12.5%) | | | 3 (12.0%) | N/A |
|  | Serology | 6 (18.8%) (GBS+) | | | 3 (12.0%) (GBS+) | N/A |
|  | PPROM | 5 (15.6%) | | | 0 | N/A |
|  | C-section (mean) | 29 (90.6%) | | | 9 (36.0%) | N/A |
| *Post-natal hospital course* | |  | | |  |  |
|  | EOS (+BCx ≤ 72h of life) | 0 | | | 0 | N/A |
|  | LOS (+ BCx >72h of life) | 1 (3.12%) | | | 0 | N/A |
|  | IVH | 3 (9.38%) | | | 0 | N/A |
|  | NEC | 0 | | | 0 | N/A |
| T1: 24-48 hours after birth; w: weeks; GA: gestational age; g: grams; GBS: gram positive beta-streptococcus; EOS: early onset sepsis; LOS: late onset sepsis; BCx: blood culture; IVH: intra-ventricular hemorrhage; NEC: necrotizing enterocolitis. | | | | | | |

| **Flow Cytometry Key Resources** | | | |
| --- | --- | --- | --- |
| **Name and fluorochrome** | **Clone** | **Manufacturer** | **Isotype** |
| BV421 Mouse Anti-human CD66b | G10F5 | BD Horizon | Mouse BALB/c IgM, κ |
| PE Anti-human CD11b | ICRF44 | Biolegend | Mouse IgG2b, κ |
| APC/Fire 750 anti-human HLA-DR | L243 | Biolegend | Mouse IgG2b, κ |
| BV510 Mouse Anti-human CD15 | W6D3 | BD Horizon | Mouse BALB/c IgG1, κ |
| APC Mouse Anti-human CD14 | M5E2 | BD Pharmingen | Mouse IgG2a, κ |
| FITC Mouse Anti-human CD33 | HIM3-4 | BD Pharmingen | Mouse IgG1, κ |
| Antibodies used for flow-cytometry analysis. | | | |

| **Table S2. Total PBMC Count (Proportion %)** | | | |
| --- | --- | --- | --- |
| **Cell Type​** | **Preterm (n = 7)** | **Full term (n = 6)​** | **Adults (n = 6)​** |
| Classical monocytes | 11683 (10.91)​ | 9041 (11.79)​ | 18027 (12.91)​ |
| Non-classical monocytes​ | 1739 (1.7)​ | 661 (0.91)​ | 3055 (2.09)​ |
| Intermediate monocytes​ | 1681 (1.5)​ | 2268 (3.23)​ | 4792 (3.36)​ |
| Cytotoxic monocytes​ | 97 (0.08)​ | 47 (0.07)​ | 1945 (1.26)​ |
| Megakaryocyte-like monocytes (MK-like monocytes​) | 177 (0.21)​ | 136 (0.24)​ | 596 (0.4)​ |
| PMN-MDSC​ | 6901 (10.56)​ | 1388 (2.56)​ | 362 (0.38)​ |
| M-MDSC​ | 3364 (4.97)​ | 1401 (1.99)​ | 2912 (2.58)​ |
| E-MDSC​ | 7851 (8.4)​ | 5444 (6.15)​ | 7378 (4.5)​ |
| Conventional dendritic cell (cDC1​) | 7 (0.01)​ | 2 (0)​ | 61 (0.04)​ |
| Conventional dendritic cell (DC2​) | 568 (0.56)​ | 435 (0.58)​ | 1642 (1.45)​ |
| Plasmacytoid (pDC​) | 109 (0.12)​ | 98 (0.15)​ | 505 (0.37)​ |
| B intermediate​ | 545 (0.57)​ | 358 (0.54)​ | 1131 (0.86)​ |
| B memory​ | 109 (0.06)​ | 90 (0.1)​ | 815 (0.57)​ |
| B naive​ | 2701 (2.93)​ | 1987 (2.81)​ | 3562 (3.48)​ |
| Plasmablast​ | 6 (0.01)​ | 0 (0)​ | 258 (0.27)​ |
| CD4 CTL​ | 0 (0)​ | 0 (0)​ | 171 (0.17)​ |
| CD4 Naive​ | 13136 (10.79)​ | 11443 (13.93)​ | 5778 (5.69)​ |
| CD4 Proliferating​ | 169 (0.22)​ | 44 (0.06)​ | 45 (0.04)​ |
| CD4 TCM​ | 40595 (21.75)​ | 45147 (34.8)​ | 26358 (22.34)​ |
| CD4 TEM​ | 358 (0.17)​ | 218 (0.16)​ | 2090 (1.54)​ |
| CD8 Naive​ | 6120 (6.06)​ | 4227 (5.66)​ | 4091 (4.23)​ |
| CD8 Proliferating​ | 9 (0.01)​ | 2 (0)​ | 11 (0.01)​ |
| CD8 TCM​ | 868 (0.55)​ | 499 (0.5)​ | 2025 (1.75)​ |
| CD8 TEM​ | 1887 (1.19)​ | 1040 (1.23)​ | 16117 (14.16)​ |
| Gama delta T-cells (γδT​) | 2338 (1.25)​ | 1887 (1.46)​ | 1801 (1.19)​ |
| Regulatory T-cells (Tregs​) | 5427 (2.66)​ | 4143 (2.9)​ | 1045 (0.96)​ |
| Mucosal-associated invariant T-cells (MAIT​) | 13 (0.01)​ | 7 (0.01)​ | 2633 (3.13)​ |
| Natural Killer cells (NK​) | 1324 (1.24)​ | 733 (1.21)​ | 8998 (6.83)​ |
| NK Proliferating​ | 163 (0.19)​ | 30 (0.06)​ | 149 (0.12)​ |
| NK_CD56bright​ | 217 (0.24)​ | 173 (0.26)​ | 434 (0.58)​ |
| Innate lymphoid cells (ILC​) | 485 (0.29)​ | 118 (0.15)​ | 117 (0.1)​ |
| Hematopoietic stem and progenitor cells (HSPC​) | 505 (0.55)​ | 245 (0.33)​ | 120 (0.12)​ |
| AXL^+^ Siglec-6^+^ dendritic cells (ASDC​) | 10 (0.01)​ | 3 (0)​ | 23 (0.02)​ |
| Eryth​rocytes | 4819 (6.81)​ | 3214 (4.51)​ | 96 (0.12)​ |
| Platelet​s | 824 (1.67)​ | 143 (0.28)​ | 830 (0.64)​ |
| **Total​** | **119128 (100)​** | **98422 (100)​** | **121701 (100)​** |
| Total cell counts (relative proportion) by age group. PBMC, peripheral blood mononuclear cells. | | | |

**Fig. 3 Supplementary Files – cannot embed excel files into Word. Please see additional files.**

**Fig. 4 Supplementary Files – cannot embed video into Word. Please see additional files.**

**
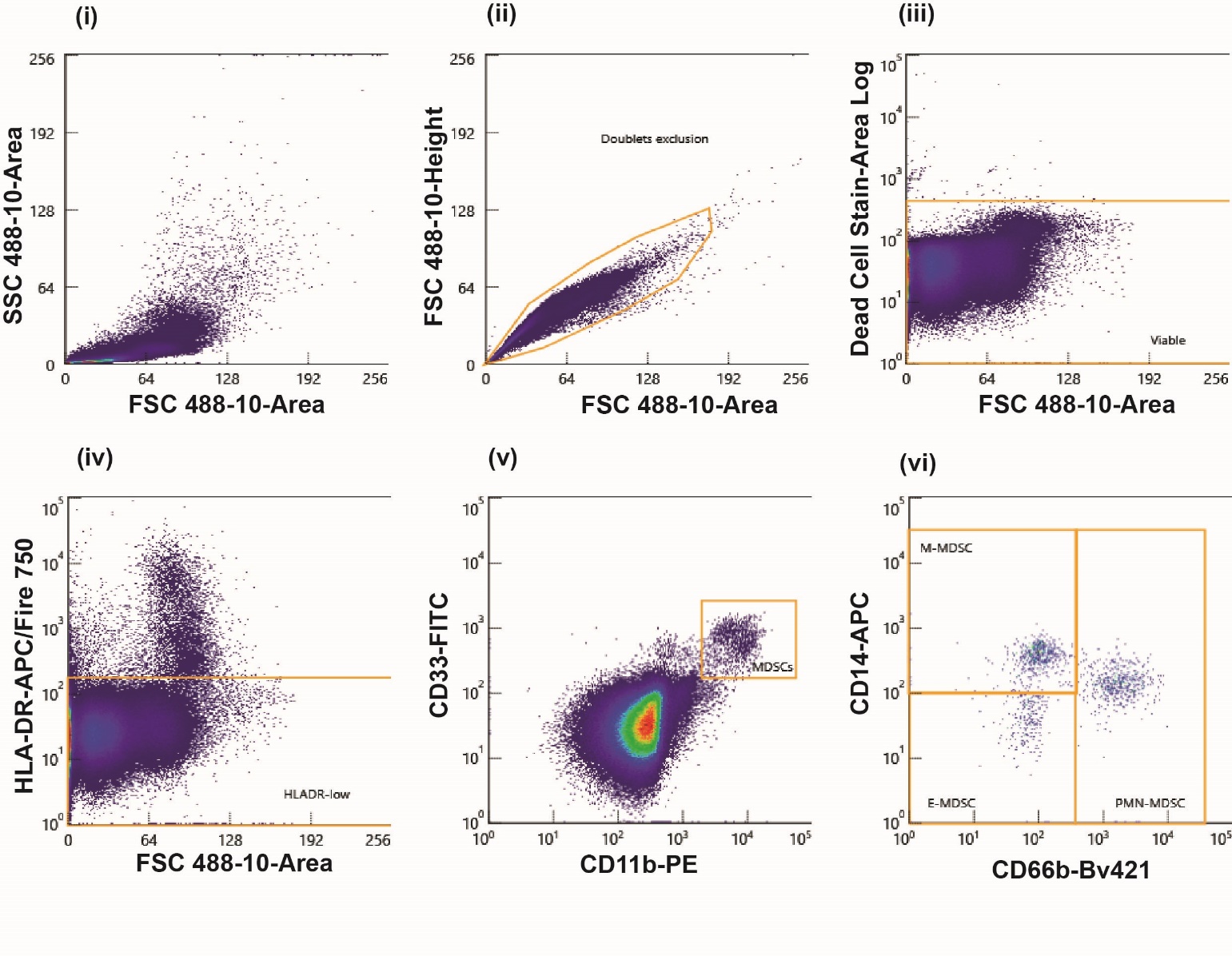
**

**Figure S1. Gating strategy for the identification of circulating MDSC subsets.** Doubles were excluded and live PBMCs were gated (i-iii). To quantify MDSCs, flow cytometry was performed on fresh PBMCs from preterm (n = 32) and full-term (n = 25) neonates, and healthy adults (n = 11). MDSCs were identified as (iv) HLA-DR^-/low^, (v) CD11b^+^CD33^+^ cells with (vi) granulocytic (PMN-MDSCs) and monocytic (M-MDSCs) subsets expressing CD66b and CD14, respectively.


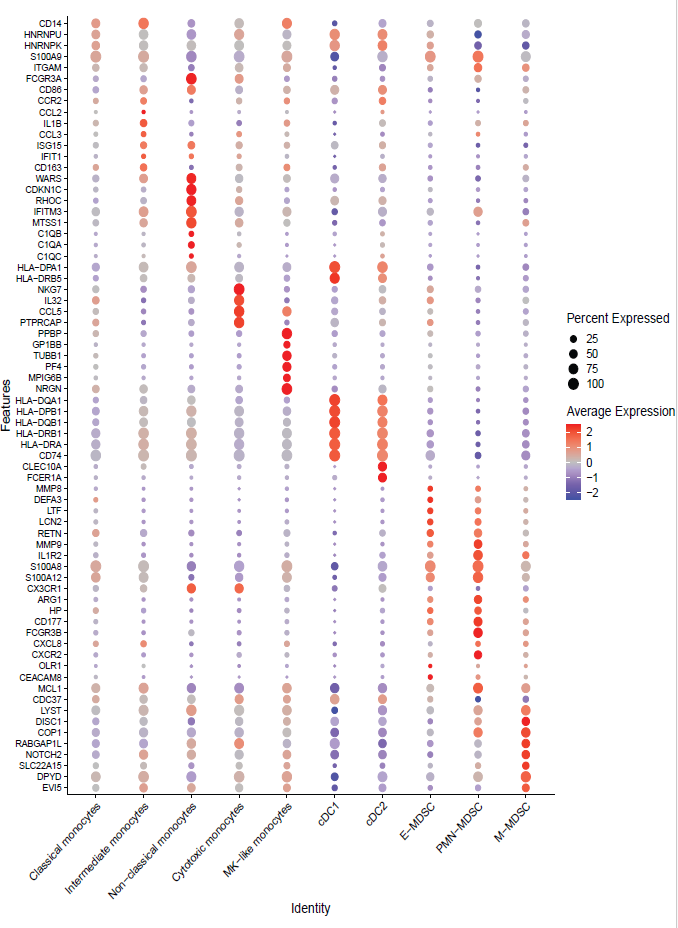


**Figure S2. Myeloid Dot plot** highlights the most significant marker genes for each cell type of the myeloid compartment. Each column represents a distinct cell type, and each row corresponds to a canonical marker gene. Dot size reflects the percentage of cells expressing the gene, and dot color indicates the average expression level within the cell type.


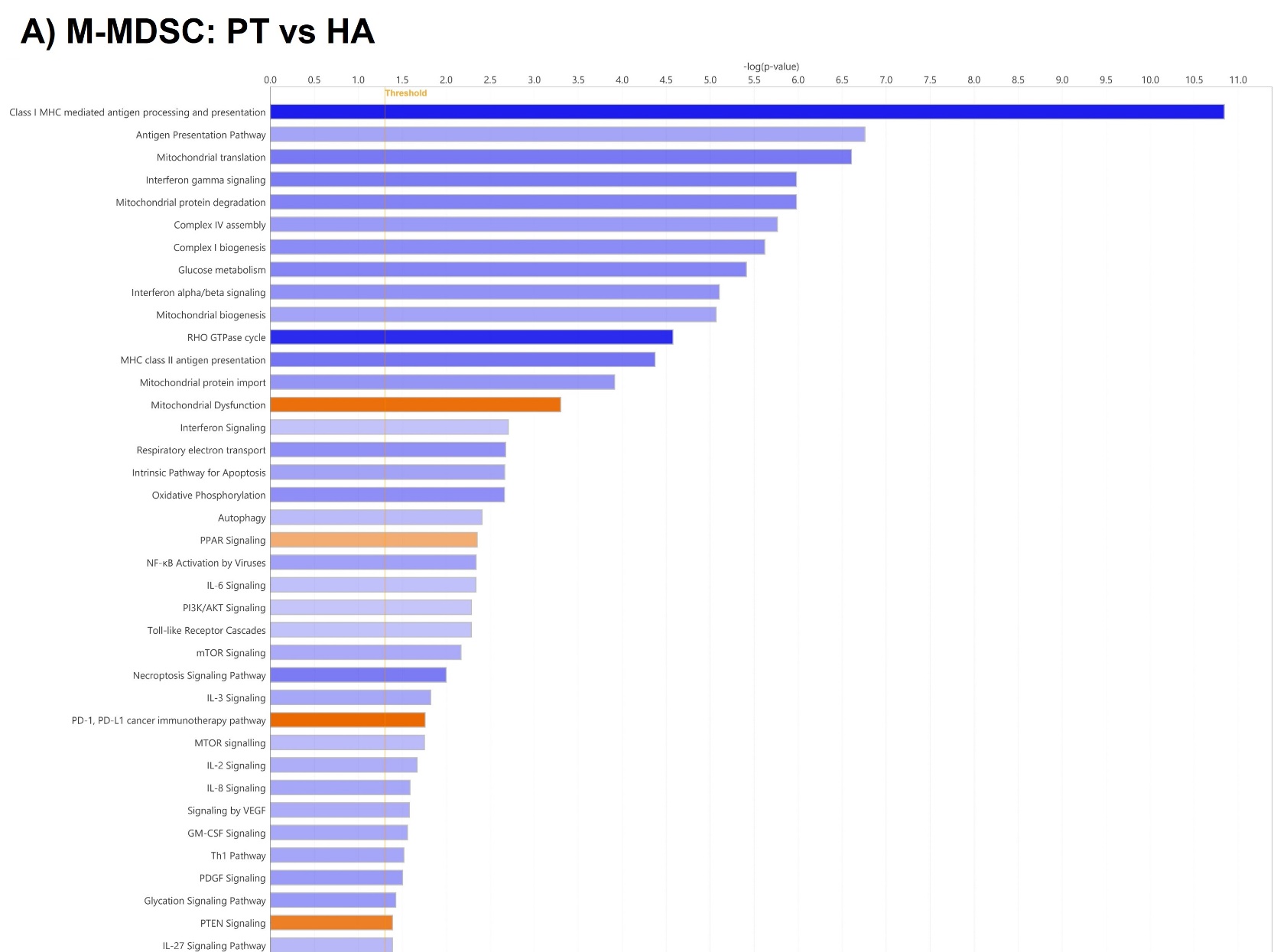


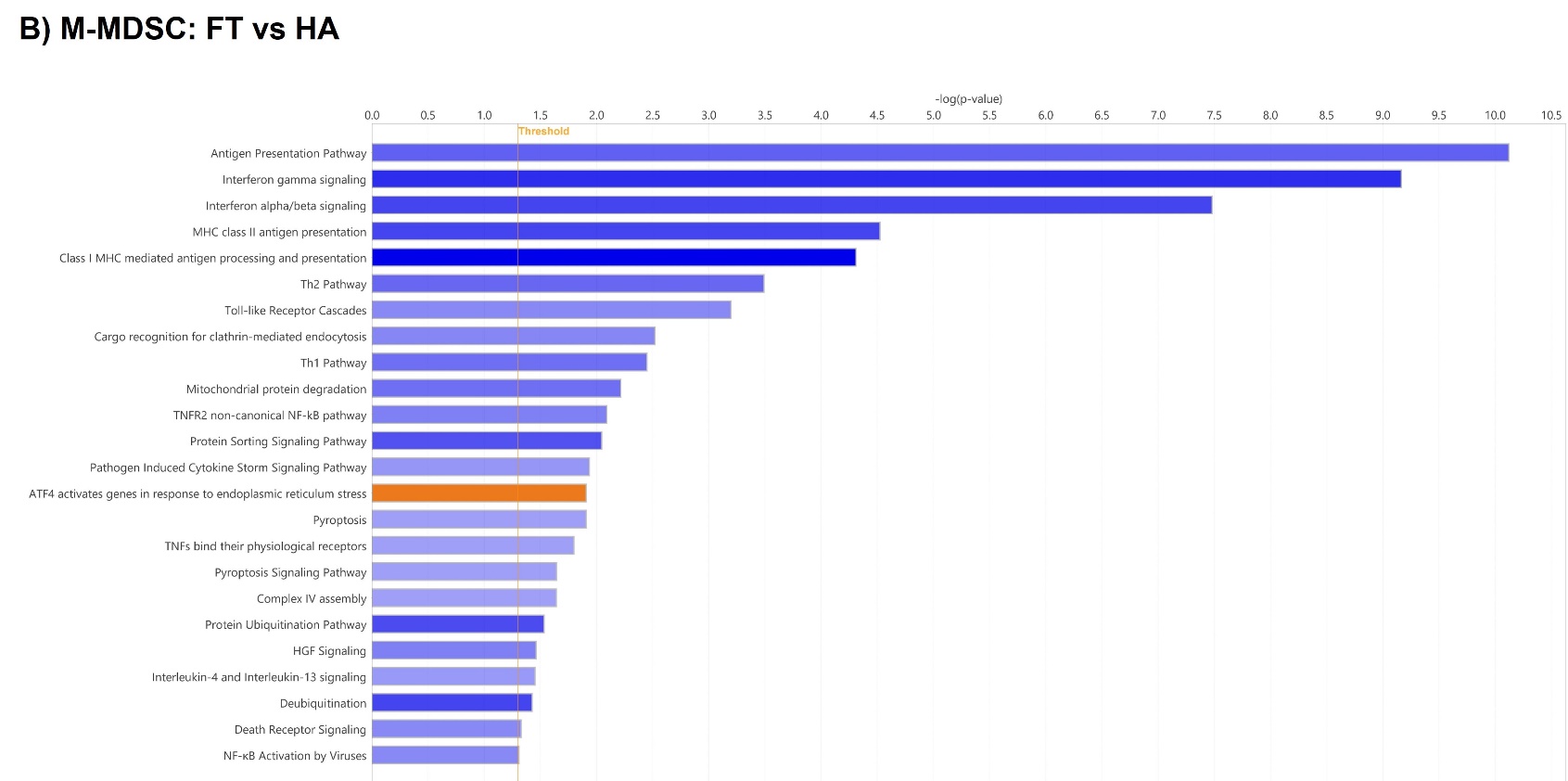


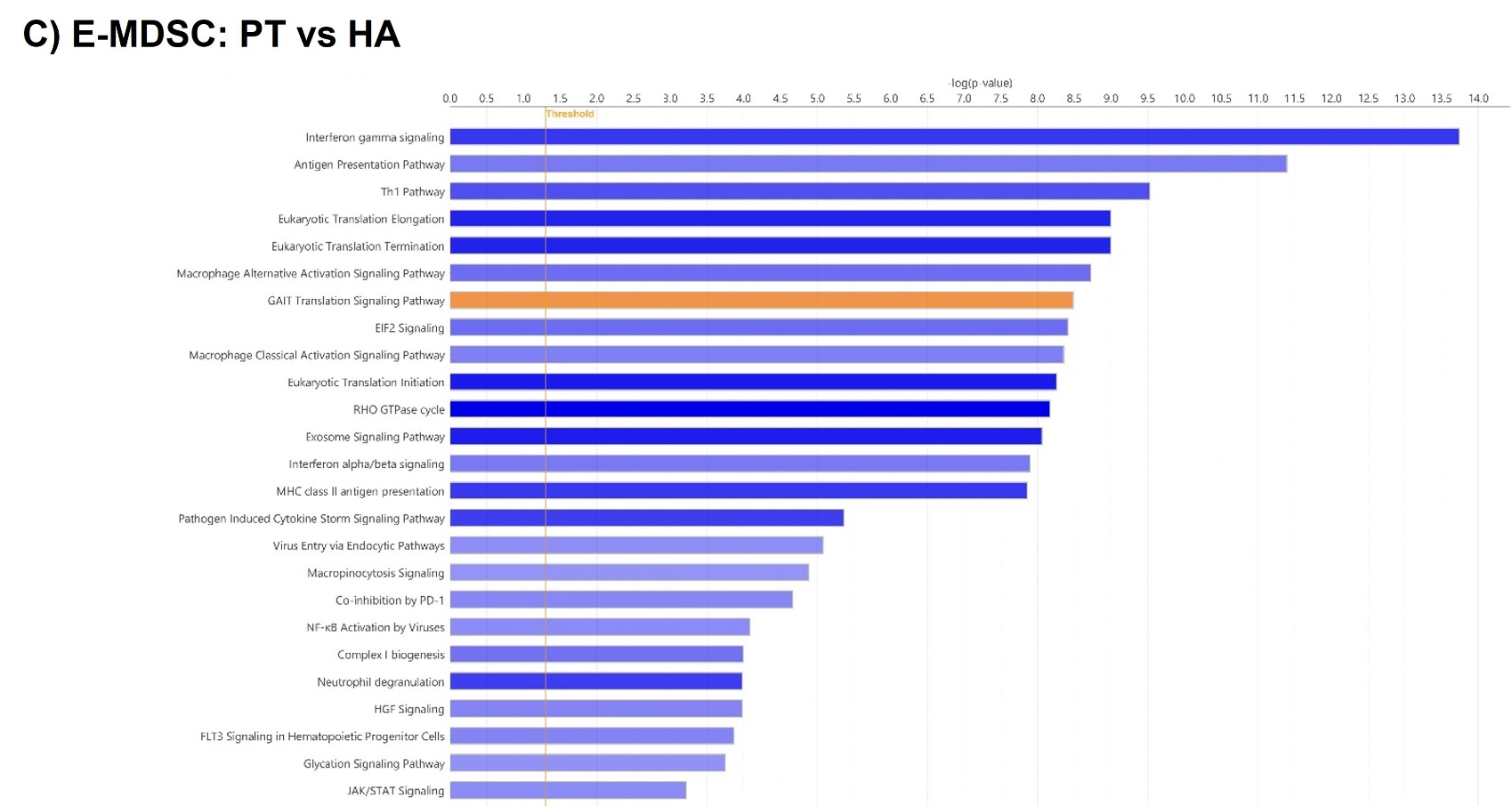


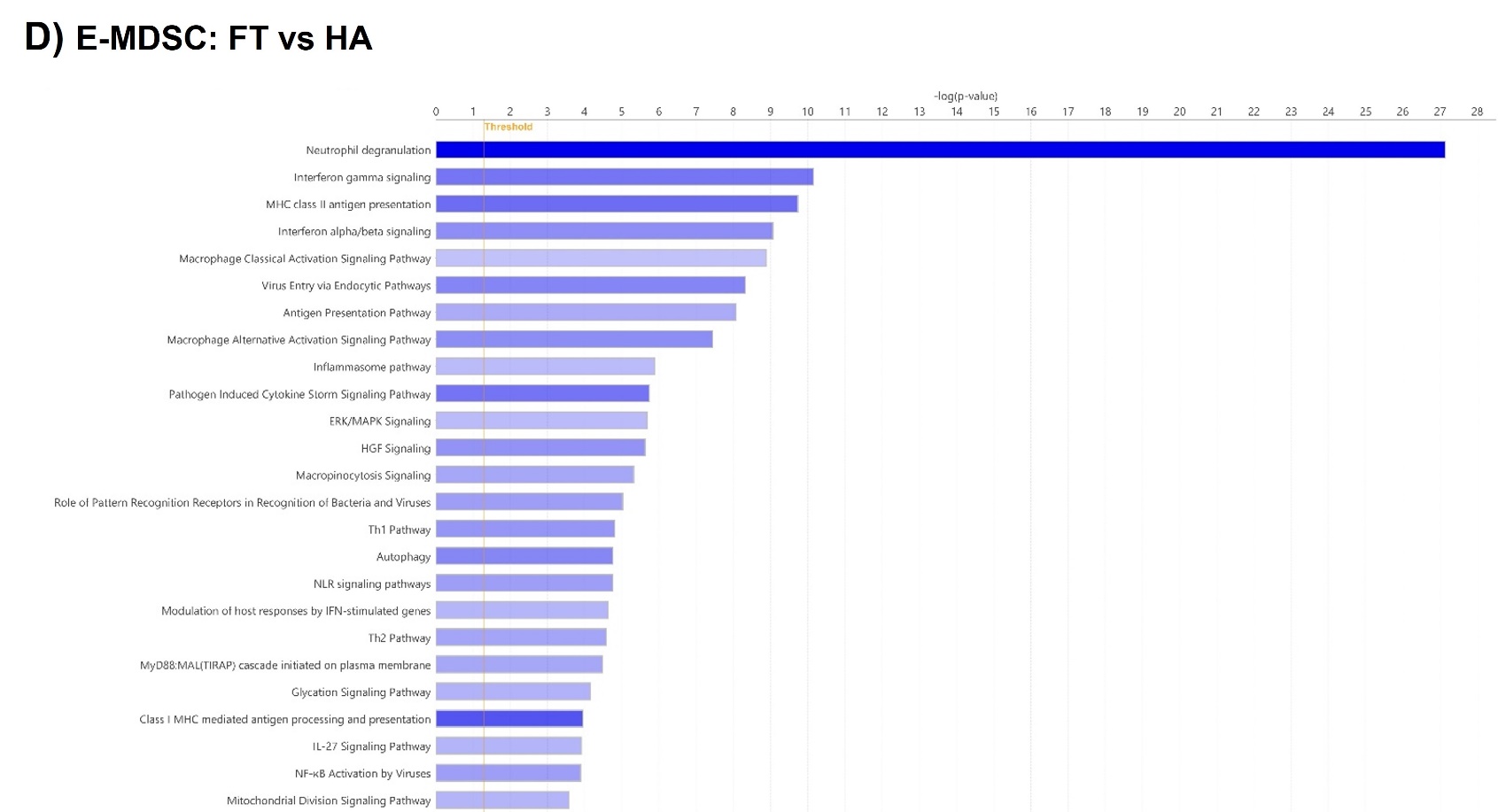


**Figure S3. Transcriptional profiling of E- and M-MDSCs reveals age-specific gene signatures and pathway enrichment. A)** Canonical pathway enrichment based on DEGs in M-MDSCs from preterm (PT) neonates compared to healthy adults (HA) and **B)** full-term (FT) neonates versus healthy adults. **C)** Canonical pathway enrichment based on DEGs in E-MDSCs from PT neonates compared to HA and **D)** FT neonates compared to HA. **(C-D)** Only demonstrate the top 25 most significant genes. Bar color indicates activation z-scores: orange (z > 2, predicted activation), blue (z < -2, predicted inhibition). Enrichment threshold: –log_10_(p-value) ≥1.3, corresponding to p <0.05.


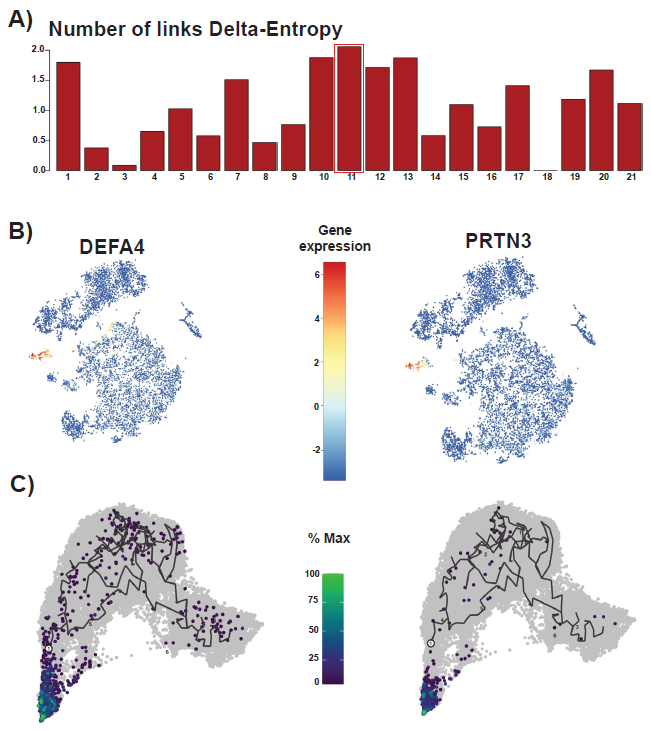


**Figure S4. Validation of trajectory root location.**
**(A)** Bar plots showing the final entropy per each of the 21 unsupervised RaceID3 cell clusters. Cluster 11 displays the highest StemID2 score, indicating the greatest differentiation potential, suggesting its role as the most upstream progenitor population. **(B)** Upregulation of the early hematopoietic stem cell progenitor marker *DEFA4 and* neutrophil-associated marker *PRTN3* are identified in our PBMC’s cluster 11. **(C)** Monocle3 pseudotime trajectory confirms upregulation of *DEFA4* and *PRTN3* at the cluster of interest (identified in **Fig. 4A-C**), corresponding to pseudotime origin of progenitor MDSCs (E-MDSCs).


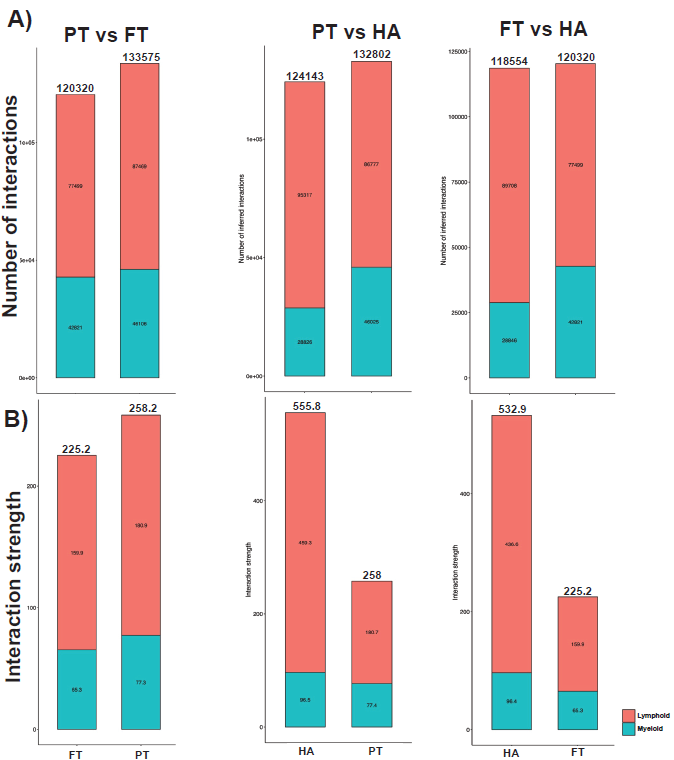


**Figure S5. Intercellular communication between MDSCs and immune effector cells is age specific.** Bar plots showing the number of ligand-receptor interactions **(A)** and interaction strength **(B)** among myeloid (teal) and lymphoid (peach) cells in preterm (PT; n = 7) and full-term (FT; n = 6) neonates, compared to healthy adults (HA; n = 6). The total interaction strength was calculated by summing the communication probability of all inferred interactions. P-values are not provided since these are inferred interactions. Overall, neonates have a greater number of interactions compared to healthy adults. The interactions strength of healthy adults is stronger than neonates. However, preterm neonates have more number of interactions and stronger signals when compared to full-term.


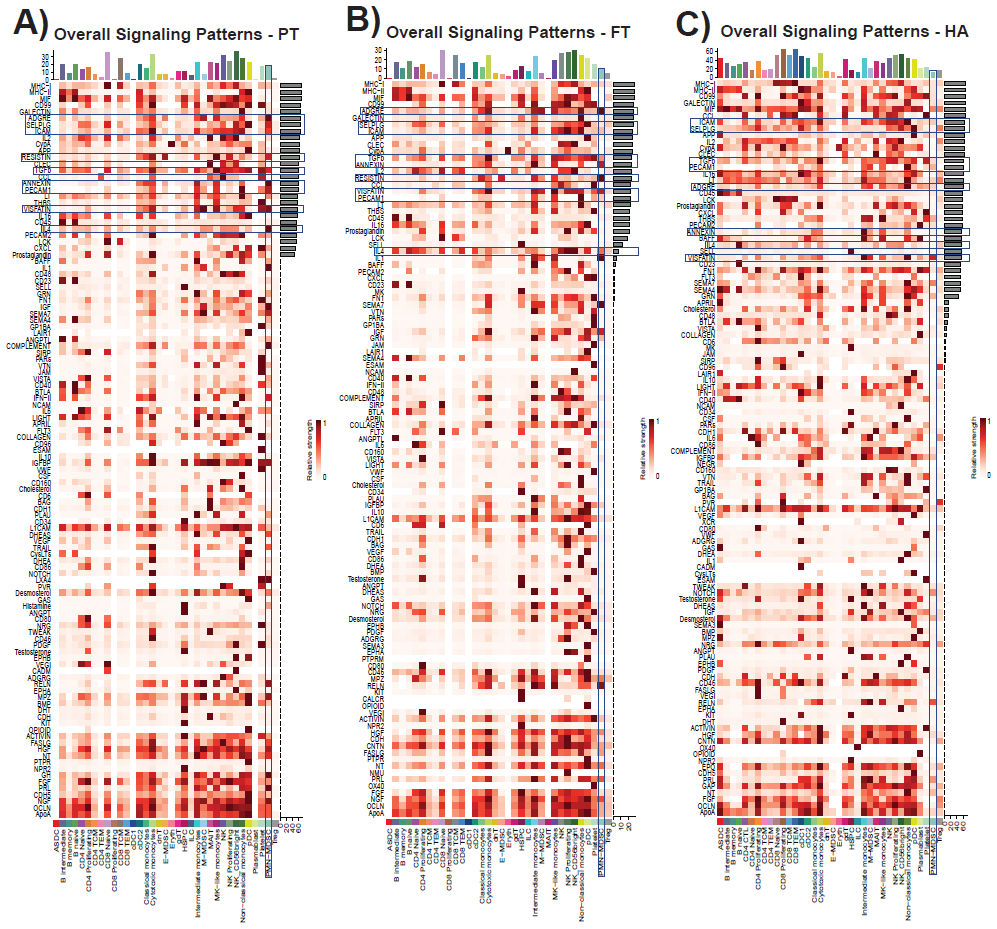


**Figure S6. Age-dependent differences in overall signaling activity and PMN-MDSC-specific ligand-receptor communication.** Heatmap showing the overall signaling patterns in preterm **(A)**, full-term neonates **(B)** and healthy adults **(C)**. The multicolored scale (top) represents the sum of incoming and outgoing signals for each respective cell. Gray bars (right) represent the sums of signaling patterns from each network and cell. ADGRE, SELPLG, ICAM, RESISTIN, TGFβ, ANNEXIN, PECAM1, VISFATIN and IL4 are preserved to have the greatest sum of patterns across age and have increased relative strength in preterm-derived PMN-MDSCs. The navy-blue rectangles are meant to guide the reader to visualize these patterns.


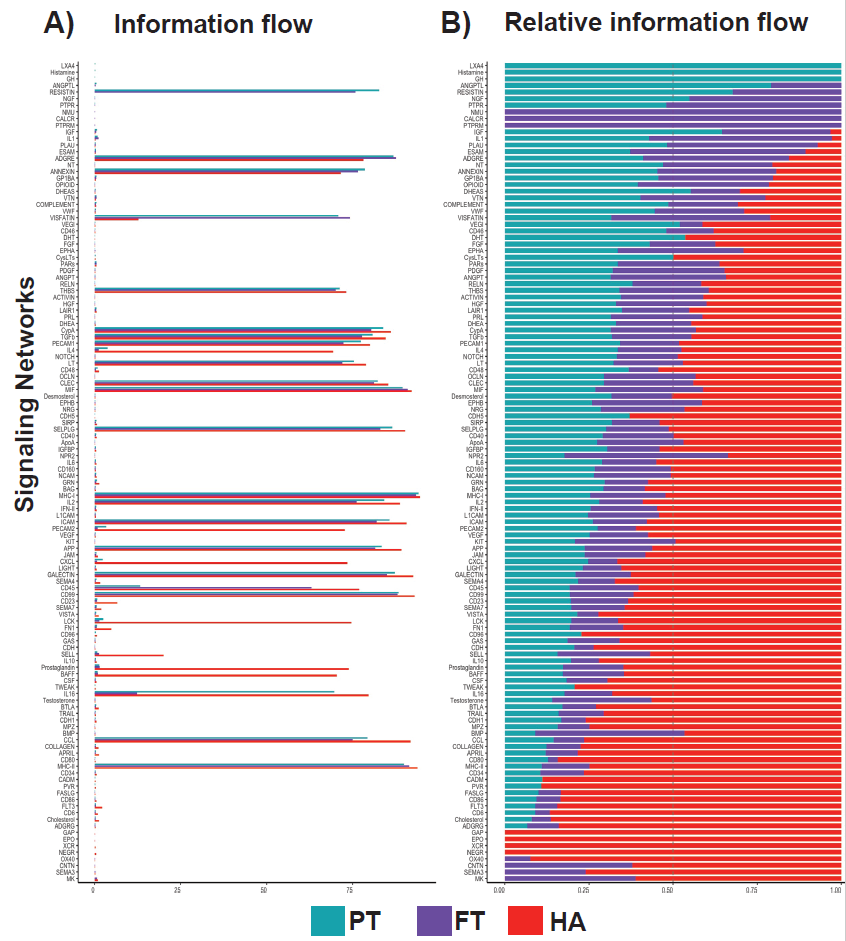


**Figure S7. Comparison of information flow across signaling pathways by age group. A)** Bar plots displaying the total information flow for each signaling pathway in preterm, full-term neonates and healthy adults. **B)** Relative contribution of each age group to the overall information flow of each signaling pathway. The stacked horizontal bars represent the proportion of information flow attributed to preterm, full-term neonates, and healthy adults for each signaling pathway, normalized to 1. For example, LXA4 seems exclusive to preterm neonate expression **(B),** but the amount of LXA4 is actually negligible compared to other pathways **(A).**
